# A new yeast strain for valorisation of vinasse, a rum distillery waste product

**DOI:** 10.1186/s13068-025-02671-0

**Published:** 2025-07-18

**Authors:** Brigita Simonaviciene, Ayokunle Araoyinbo, Juwayria Ali, Jamie McGowan, David A. Fitzpatrick, Gary Jones, Celia Ferreira, Andrew R. Pitt, Corinne M. Spickett, Vincent Postis, Carine de Marcos Lousa

**Affiliations:** 1 Centre for Biomedical Science Research, School of Health, Leeds Beckett University, Biomedical Sciences, Leeds, LS13HE UK; 2 UCD School of Biomolecular and Biomedical Science, University College Dublin, Belfield, Dublin 4, Ireland; 3 Department of Biology, Maynooth University, Co. Kildare, Maynooth, Ireland; 4 School of Food Science and Nutrition, University of Leeds, Leeds, LS29JT UK; 5 Manchester Institute of Biotechnology, University of Manchester, Manchester, M1 7DN UK; 6 School of Biosciences, Aston University, Aston Triangle, Birmingham, B4 7ET UK; 7 Aston Institute for Membrane Excellence, Aston University, Birmingham, B4 7ET UK; 8 Present Address: Drug Discovery Unit, University of Dundee, Dundee, DD15EH Scotland; 9 Present Address: Centre for Plant Sciences, University of Leeds, Leeds, LS29JT UK; 10 Present Address: Centre for Integrative Genomics, Faculty of Biology and Medicine, University of Lausanne, Lausanne, Switzerland

**Keywords:** Vinasse, *Pichia kudriavzevii*, *Yarrowia lipolytica*, Biofuel, Rum distillery

## Abstract

**Background:**

Waste valorisation refers to processes of reusing or recycling waste materials to create valuable products. In the Rum distillery industry, the primary waste byproducts include bagasse, a solid waste made up of sugar cane residue and vinasse, a thick and acidic liquid. Although vinasse has been repurposed in agricultural fields, it has also contributed to both soil and ocean pollution. Despite several potential solutions having been suggested, an effective and environmentally safe use for vinasse has yet to be found.

**Results:**

The valorisation of vinasse for biofuel production was explored by assessing its potential as a growth medium for lipid production by non-conventional yeasts. The oleaginous yeast strain *Yarrowia lipolytica*, known for its lipid production capabilities, was initially tested on vinasse but required further adaptation and optimization. To circumvent this, we isolated a novel yeast strain from old vinasse waste, named V1, which demonstrated strong growth potential. The growth conditions of V1, including temperature and acidity, were characterized, and its potential for bioengineering was evaluated. This strain exhibited resistance to highly acidic pH levels and higher temperatures when cultivated on YPV, an artificial laboratory medium designed to mimic the acidity and glycerol content of vinasse. Whole genome sequencing (WGS) identified V1 as *Pichia kudriavzevii*. We demonstrated that V1 could be transformed with *Yarrowia lipolytica* vectors using the classical yeast heat shock protocol, thus enabling potential genetic engineering. Finally, lipid content in V1 was analysed in different conditions, confirming the strain's potential for biofuel production.

**Conclusions:**

*Pichia kudriavzevii* is not a traditional yeast, but its ability to adapt and grow under extreme pH and higher temperature conditions makes it a promising candidate for rum industry waste management applications. This strain could potentially be utilised to convert vinasse and other food waste products into valuable biofuels. Although further research is required to engineer and optimize this novel strain for vinasse cultivation, our findings highlight its great potential as a micro-factory in rum-producing regions and high locations, where agricultural waste is in need of valorisation solutions.

**Supplementary Information:**

The online version contains supplementary material available at 10.1186/s13068-025-02671-0.

## Background

As the world production of agricultural products increases, stakeholders are faced with the difficult task of safely discarding agricultural waste products. These include fruit overproduction (mango, cocoa beans, bananas, etc*.*) as well as side products from this production, such as mango peel, seeds or kernels; cocoa honey, pod husk, etc. and many scientists are looking at ways to add value for these secondary products [1–3]. Another important source of agricultural crop waste originates from processing by distilleries, which are amongst the most polluting industries [4]. The resulting fermentation process generates large quantities of solid and liquid waste with high concentrations of organic matter. One such industry is the sugarcane rum distillery, which by a multi-step process, produces two types of waste: bagasse, which is the dry fibrous residue generated when extracting the juice from the sugarcane; and vinasse which is produced as a final by-product of the distillation process. For every litre of rum produced, between 10 and 15 L of vinasse is generated as a by-product [5]. Historically, a common practice in countries producing ethanol from sugarcane was fertigation of crop fields with these high amounts of vinasse. Unfortunately, widespread uncontrolled fertigation has led to growing ecological concerns on the detrimental impact of such practice on soil quality and aquatic life [6–12]. Several researchers have conducted studies to find more sustainable treatment methods for circular agriculture. These methods include controlled fertigation, concentration of vinasse via evaporation, poultry and livestock feed, compost or combustion and anaerobic digestion [13–17]. However, due to an acidic pH and high concentrations of residues and minerals present in vinasse, each method comes with its own challenges and drawbacks. Indeed, the composition of vinasse varies depending on the crop source, the soil and the distillation process [16]. A better understanding of the complex composition of vinasse from various sources and its high nutrient content has led researchers to suggest vinasse as a microbiological growing medium for the production of secondary metabolites with added value [18–21]. Despite a number of microorganisms having the ability to grow on vinasse, including bacteria, such as *Pseudomonas aeruginosa* and various fungi, the translation into the production of biofuels has not yet been successful [21–25].

Therefore, the aim of this study was to investigate new ways to add value to vinasse using it as a culture medium for the production of lipids by engineered conventional or non-conventional yeasts. We explored various methods to isolate enhanced mutants of classical strains or identify new strains able to grow on vinasse. We report here the identification and characterisation of a novel yeast strain with enhanced growth properties compared to traditional yeast strains.

## Materials and methods

### Yeast media

Yeast strains were grown on either Yeast Peptone Dextrose (YPD) (1% yeast extract, 1% peptone, 2% glucose) or a laboratory designed “Yeast Peptone Vinasse” (YPV) (1% yeast extract, 1% peptone, 5% glycerol, and pH 4.85) to mimic high glycerol content and low pH of vinasse. For the preparation of plates, 2% agar was added to the medium before autoclaving. For the preparation of media at acidic pH, YPD was adjusted to acidic pH first. The adjusted YPD and agar solutions were then autoclaved separately and mixed just before pouring into the petri dishes, to avoid agar degradation. When appropriate, 300 µg/mL of either hygromycin, gentamicin or nourseothricin was used.

Vinasse samples for this study were collected from Longueteau Distillery in Guadeloupe (French Caribbean). Two samples were obtained: one “fresh” vinasse collected just after distillation and one matured vinasse collected a few months after distillation and stored in at + 4 °C. Prior to use in culture media, freshly acquired vinasse was centrifuged thrice at 4000*g* for 10 min to remove debris. For selection of the vinasse microbiome, 1 mL of centrifuged vinasse was spread onto YPD plates. For growth curves, vinasse was centrifuged and filtered through a 0.45 µm filter, diluted 1:1 with distilled water and autoclaved at 121 °C for 20 min. The measured pH of fresh vinasse was 3.6 and mature vinasse 4.2.

### Yeast strains

Various strains were used in this study and summarised in Table 1.

**Table 1:** List of yeast strains used in this study

Yeast strain	Name in this study	Type/genotype	Refs.
*Saccharomyces cerevisiae* JL1-3	Sc	MATα *ade2-1, leu2-3,112, his3-11,15, trp1-1, can1-100, ura3-1, anc1::LEU2, anc2::HIS3, anc3::URA3*	[26]
*Yarrowia lipolytica*	YL	Type ISA 1718 T	[27]
*Pichia kudriavzevii* (commercial)	PK	ASM198332v1 wild-type	PYCC
*Pichia kudriavzevii* (isolated on vinasse)	V1	This study	This study

PYCC refers to the Portuguese Yeast Culture Collection

### Yeast growth

Yeast liquid growth was performed as followed: a freshly grown yeast colony was inoculated in 5 mL YPD or YPV and grown overnight at 28 ℃ in a shaking incubator 200 rpm with a minimum liquid/air ratio of 1:10. The overnight preculture was used to inoculate a new culture in a 250 mL baffled flask at 0.1 OD and then grown at various temperatures at 200 rpm in an Innova New Brunswick incubator. The OD of the culture was monitored every 2 h by taking an aliquot in sterile conditions and measuring the optical density OD_600nm_ in a spectrophotometer (Biochrom WPA CO8000 Cell Density Metre). Dilutions 1:100 in similar media were made when OD was higher than 1.

### Measuring the cell count/ml/OD and drop tests

The relationship between OD and cell count was established for yeast strains V1, PK, YL or Sc.

YPD media (10 mL) was inoculated with either PK, V1, YL, Sc in 50 mL Falcon tubes and incubated in a shaker overnight at 28 °C. After 24 h, the OD was measured at 600 nm and an aliquot of the culture was diluted to a final OD_600nm_ of 0.1. A further serial dilution of 1:10 was made. An aliquot of 10 μL from each tube was spread onto YPD plates and incubated for 48 h at 28 °C. After 48 h, the colonies were counted and cell count/mL/OD was calculated. This allowed the optimal dilutions for drop tests to be established. Similarly, a dilution of the overnight preculture was performed to get a final OD_600_ of 1 in a 1 mL final volume, which corresponds to 10^6^ cells/ml. A serial dilution 1:10 was then performed and a 10 μL aliquot from every dilution was pipetted onto the appropriate agar plate. The plate was then incubated for various times (24 h or 48 h) at various temperatures (28 °C up to 45 °C).

### Yeast transformation

For the transformation, the previously published plasmid developed for *Yarrowia lipolytica* was used. The plasmid contained a cassette of genes for the production of β-carotene under the control of the TEF promoter [28]. Prior to transformation, the plasmid was digested by *Sfi*I and 1 µg of the total digest was used for transformation. Yeast transformation was performed by adapting a previously published protocol [29]. Yeast cells were grown overnight in YPD media. The next day, 50 mL of YPD media, prewarmed at the temperature of desired growth, was inoculated to an OD_600_ of 0.1 and incubated at 200 rpm until the OD was 0.2–0.4. The cells were collected by centrifugation at 3000 g for 5 min. The cell pellet was washed three times with 25 mL of sterile water. The cells were resuspended in 1 mL, then transferred into a 1.5 mL microcentrifuge tube, centrifuged for 30 s at 13,000*g* and washed twice with distilled water. 100 μL cells were aliquoted for each transformation and 360 μL of transformation master mix (240 μL PEG 3350 (50%w/v), 36 μL Lithium Acetate 1 M, 50 μL single stranded carrier DNA (2 mg/mL), 1 μg linearised DNA) was added to each tube. The resulting 460 μL mixture was homogenised and incubated at 30 °C for 30 min in a shaking incubator. The cells were subjected to a heat shock at 42 °C in a water bath and incubated for 15 min. After the incubation, the cells were either pelleted and resuspended in 1 ml of sterile media, or the mix was incubated for a further 2–3 h at 28 °C in a shaking incubator set to 200 RPM prior to spreading on antibiotic containing plates. Plates were incubated for 3–4 days at 28 °C.

### Biomass measurements

The biomass was measured by washing cells with distilled water twice, followed by centrifugation at 3000*g* for 5 min. The cell pellet was oven dried at 95 °C for 24 h. The dry biomass was weighed in grams.

### Lipid extraction

Extraction of yeast lipids was performed following the protocol of Schneiter & Daum [30]. Yeast cells were grown as described above in 100 mL of media to an OD_600_ of 0.1 in a 500 mL baffled flask at either 28 °C or 37 °C. The cells were harvested by centrifugation for 10 min at 3000*g* and washed with double-distilled water. The cell pellet was weighed and then mixed with 10 mL of methanol and transferred to a glass bottle. To disrupt the cells, 20 g of acid-washed glass beads were added to the solution and vortexed for 4 periods of 30 s each followed by 30 s cooling on ice. Chloroform (20 mL) was then added to give a 2:1 (v:v) ratio of chloroform:methanol and shaken at 200 rpm for 1 h at room temperature. The samples were filtered using a sintered glass funnel and transferred into a glass beaker. 10 mL of 0.034% MgCl_2_ was added, and the mixture was shaken for 15 min before centrifuging at 3000*g* for 5 min. The upper layer was carefully aspirated, and the remaining organic phase was washed with 10 mL of 2N KCl:methanol (4:1; v:v) and re-centrifuged. The upper layer was removed again, protein at the phase boundary was removed and the remaining organic phase was washed sequentially with 10 mL of 3:48:47 chloroform:methanol:water until the phase boundary became clear. The final organic phase was stored − 20 °C until analysis.

### Lipidomic analysis by LC–MS/MS

Aliquots (5 µL) of triplicate samples of YL, V1 (grown at 28 °C or 37 °C) were diluted in 95 µL of the chromatography solvent A described below and analysed by liquid chromatography-MS^e^ on a Waters I-class UPLC connected to a Waters SELECT Series Cyclic IMS. 5 µL of diluted lipid extract was injected onto an Accucore™ C30 reverse phase column, 150 × 2.1 mm (length x internal diameter), 2.6 µm particle size (ThermoScientific, UK) at a flow rate of 200 µL/min and temp of 50 °C. Lipid separation was achieved using a 30 min gradient of the following mobile phases: 50:50 acetonitrile:H_2_O containing 10 mM ammonium formate and 0.1% formic acid (Solvent A) and 85:10:5 isopropanol:acetonitrile:H_2_O containing 10 mM ammonium formate and 0.1% formic acid (Solvent B). The gradient was formulated as follows: starting solvent 10% B ramping to 30% at 4 min, 40% B at 5 min, 90% B at 20 min, 99% B at 21 min held until 24 min, then decreasing to 10% B at 25 min and equilibrated until 30 min. For the mass spectrometry, separate runs for positive and negative ion modes were used. The parameters for positive ion were capillary voltage 2.0 kV; cone voltage 40 V; source offset 20 V; source temperature: 120 °C; desolvation temperature 280 °C; cone gas 30 L/h; desolvation gas 600 L/h. For negative ion the same parameters were used, but with the opposite voltage polarities. Scans were alternated between low (6 V) and high (25–60 V ramp) collision energies in the transfer cell to acquire MS^only^ and MS^e^ scans, respectively. The m/z range for both ionization modes was 100–2000 Da with a scan time of 0.5 s. Lockmass data were collected using LeuEnk infusion at 10 μL/min and a lockmass spectrum was collected for 0.2 s at a 1-min frequency.

### Lipidomic data analysis

LC–MS/MS data were imported into Progenesis QI (Nonlinear Dynamics, UK) for chromatography alignment and identification of LC–MS features/compound ions. Global normalization was used for quantification. Adduct selection for positive ion mode was M + H, M + 2H and M + NH_4_ and for negative ion mode M-H, M-2H, M + Na-2H, M + FA-H and M-H_2_O -H, where FA is formic acid. Default parameters were used for other settings. The mass spectra for the features were searched against the Yeast Metabolome Database (http://www.ymdb.ca/) using the Progenesis MetaScope algorithm and identified by matching accurate mass, and where present MSMS data, to the theoretical database. In positive ion mode 10,453 compound ions were detected, from which 331 compounds were identified by database matching. The list of identified compounds was further refined by excluding compounds with a mass-to-charge ratio < 350 Da, a retention time of < 5 min and a mass error > 5 ppm. The remaining features were then examined manually for appropriate chromatographic separation, isotope selection and fragmentation data. Where it was not possible to differentiate between isomers, either all possible species or the total carbons and double bonds are reported. Identification of features was also checked by data matching to the LIPID MAPS structural database.

The data were also analysed manually in MassLynx software to investigate levels of lipids containing specific fatty acids or phosphocholine. Extracted ion chromatograms were generated from the negative ion high energy MS^e^ data for a range of expected fatty acid fragment masses (C16:1, 253.22; C16:0, 255.23; C18:3, 277.21; C18:2, 279.22; C18:1, 281.24; C18:0, 283.25; C20:1, 309.28; C20:0, 311.29). Peaks in the chromatograms were integrated using the MassLynx software and the proportion of the free fatty acid was calculated as a percentage of the total amounts of lipids containing that fatty acid. For phosphatidylcholines, extracted ion chromatograms were generated from the positive ion high energy MS^e^ data for the fragment mass 184.07, corresponding to phosphocholine.

### Microbiome analysis

Microbiome analysis of fresh and mature vinasse was performed by Eurofin genomics using standard amplicon libraries used by the provider and described in https://eurofinsgenomics.eu/en/eurofins-genomics/material-and-methods/microbiome-sequencing/.

### Whole genome sequencing

Whole genome sequencing (WGS) was performed by Eurofin genomics using the Illumina platform, the resultant reads were paired end and 150 bp in length. Reads were trimmed with TrimGalore (https://github.com/FelixKrueger/TrimGalore).

The trimmed reads were aligned to the reference *Pichia kudriavzevii* str. 129 genome (Accession: GCA_001983325.1) using BWA MEM (ver. 0.7.17) [31]. SNPS were called based on the GenomeAnalysisToolkit (GATK) (ver. 4.3.0.0) Best Practices Workflows (https://gatk.broadinstitute.org/hc/en-us/sections/360007226651-Best-Practices-Workflows) [32]. In brief, aligned BAM files were sorted and duplicate reads were marked using GATK SortSam and MarkDuplicates tools, respectively [32]. Variants were called with GATK HaplotypeCaller Variant files were filtered using GATK VariantFiltration with the following cutoffs *“-filter "QD* < *2.0" -filter "FS* > *60.0" -filter "MQ* < *40.0" -filter "SOR* > *4.0" -filter "MQRankSum* < *-12.5" -filter "ReadPosRankSum* < *-8.0*". GATK SelectVariants was used to extract the resultant filtered SNPs. SnpEff was used to annotate and predict the effects of SNPs (PMID: 22728672).

GO annotation was carried out using InterProScan [33]. Enrichment analysis of GO terms was carried out by performing a Fischer’s exact test analysis with parent term propagation and false discovery rate correction (*p* < 0.05) using the Python package GOAtools [34]. False discovery rate correction was applied for all Fischer’s exact tests in GOAtools using a *p* value distribution generated from 500 resampled *p* values. The raw V1 reads for this study have been deposited to NCBI GenBank BioProject accession number PRJNA1254219 and the SRA database with the accession SRX28499329.

### Statistical analysis

Statistical analysis of lipidomics data was performed using R (v4.x) in RStudio (version 2023.06.1 + 524; Posit Software, PBC). Fatty acid abundance data was analysed using Welch’s two-sample *t* tests to compare differences between conditions. Raw *p* values were reported for each comparison. Statistical significance was determined at a threshold of *p* < 0.05.

## Results

### Strain selection for improved growth

The composition of sugarcane vinasse is typically reported as highly acidic (between 3.5 and 5), with high mineral content and high glycerol content [8]. In particular, the glycerol content can represent 38% of sugarcane juice and 2.8–5.9% of final vinasse [24, 35, 36]. To select yeast strains with improved growth on sugarcane vinasse, we have designed a media mimicking vinasse in terms of low pH and high glycerol concentration called YPV (yeast-peptone-vinasse) as the first step to start the screening process. Since the range of pH and concentration varied depending on the type and origin of vinasse, an average value of pH 4.8 and 5% glycerol was chosen. This is relatively different from the classical YPG (Yeast-peptone-glycerol) media which contains only 2% glycerol and has a more neutral pH. We employed two parallel approaches: one aiming at selecting mutants of classical laboratory yeast strains with enhanced growth on vinasse—and the additional advantage of being amenable to genetic engineering, while the second option explored isolating novel yeast strains with better growth phenotype on YPV. For the first approach, random mutagenesis of classical laboratory yeast strains and selection of colonies on YPV was first attempted. Two yeast strain types were selected for this random mutagenesis: *S. cerevisiae* is a classical strain that can be manipulated genetically to improve growth on high glycerol content [37–39]*.* Alternatively, *Y. lipolytica* is a well-established strain for its efficient growth on glycerol and has the additional advantage of being able to synthesise lipids for biofuel production [40–43]. Both *S. cerevisiae* and *Y. lipolytica* strains were submitted to either a chemical treatment with EMS (ethyl methanesulfonate) or ultraviolet light mutagenesis and the growth tested on YPV agar plates. As expected, *S. cerevisiae* exposure did not lead to mutants with significantly improved growth on YPV (not shown), since improved glycerol consumption is achieved more efficiently by genetic manipulation [37–39]. In contrast, *Y. lipolytica* generated several mutant colonies appearing to have better growth than the untreated wild-type *Y. lipolytica* (data not shown). These colonies were isolated and the three most performing colonies (with highest growth on YPV plates) were selected for further studies on vinasse and named 5H, 6A and 9H.

In parallel, a second approach was to select yeasts directly from the vinasse microbiome. This approach has the advantage of selecting yeasts that are already growing on vinasse, but with possible limited engineering opportunities if no toolboxes are available for these alternative yeast strains, such as those available for *S. cerevisiae* and *Y. lipolytica*. A sample of two vinasse aliquots, one from a freshly produced vinasse and one from a matured vinasse (1 year), were spread onto YPV plates. A few colonies were observed for both samples, and the best growing colony (named V1) was selected (from the mature sample) for further studies.

To characterise these strains further, their growth on liquid YPV culture, their biomass and total lipid contents were first evaluated. None of the *Y. lipolytica* clones (5H, 6A and 9H) showed significant growth improvement in liquid YPV cultures (in terms of saturation and doubling time) or in biomass and total lipid production compared to wild-type YL (WT) (Table 2).

**Table 2:** Comparison of liquid growth on YPV at 28 °C

Yeast strains	Saturation OD	Doubling time (h)	Biomass (g/L)	Total lipid content (g/L)
YL	56.0 ± 4.3	2.2 ± 0.1	19.20 ± 4.67	6.42 ± 0.97
5H	58.3 ± 4.0	1.95 ± 0.05	18.74 ± 5.62	5.62 ± 0.04
6A	62.3 ± 2.1	3.1 ± 0.3	19.95 ± 3.52	7.90 ± 1.35
9H	59.7 ± 4.2	2.4 ± 0.2	26.01 ± 3.89	9.25 ± 0.63
V1	143.3 ± 32.1	2.2 ± 0.5	30.70 ± 7.78	13.80 ± 3.65

Wild-type Y. lipolytica (YL) and mutants (5H, 6A, 9H), as well as the V1 strain isolated from vinasse. Biomass and total lipid amount are also stated for each strain.

In contrast, the V1 strain showed significantly higher saturation OD, despite a similar doubling time compared to the wild-type *Y. lipolytica*. In addition, a slight increase in biomass and a doubling of the total lipid content were also observed compared to the WT. Given the enhanced potential of this strain to grow on liquid YPV, we performed a whole genome sequencing of V1 and identified V1 as being *Pichia kudriavzevii*.

### Vinasse microbiome

To further analyse the overall microbial composition of both vinasse samples and possibly identify other fungal and bacterial strains, a microbiome analysis was performed on both samples.

The yeast profile was found to be different in fresh vs mature vinasse. While *S. cerevisiae* species represented 65.6% of fungi in the fresh vinasse sample, as expected due to their fermenting capacity, *Pichia* species represented more than 80% of the fungi in mature vinasse, with the oleaginous *Pichia manshurica* and *Pichia ethanolica* being the most represented, while *Pichia kudriavzevii* only represented 0.2% of the fungi population in our sample (Table S1). Nevertheless, *Pichia kudriavzevii* has been identified in many agricultural wastes [44–47]. In particular, a *P. kudriavzevii* strain isolated from rotten fruits has been shown to produce a dry biomass and lipid yield of 33 g/L and 23% w/w, respectively, when grown on glycerol as the sole carbon source [48]. Considering the increased interest and recognition of this strain for its biotechnology and biofuel production potential, this prompted us to further characterise V1 [48–52].

### Growth of the V1 strain on vinasse

Given the strong potential of the vinasse-isolated strain V1 (*Pichia kudriavzevii*) to efficiently grow on solid and liquid YPV, we next examined the growth of this strain on vinasse. Vinasse is a very viscous medium needing dilution prior to use. Fadel et al*.* (2014) have shown that 50% vinasse in growth media was optimum for ethanol production after fermentation by *S. cerevisiae* [53]. A 1:1 dilution was, therefore, tested to compare the growth of *Y. lipolytica* (YL), the vinasse-isolated *P. kudriavzevii* (V1) strain and a commercial *P. kudriavzevii* (PK) from the Portuguese Yeast Culture Collection (PYCC). Two temperatures were tested, 28 ºC and 37 ºC. As expected, due to vinasse being more stringent than traditional media, all strains show strong growth reduction in vinasse compared to YPV (compare Tables 2 and 3, 28 ºC).

**Table 3:** Comparison of liquid growth on vinasse at two temperatures

Yeast Strain	28 ºC		37 ºC	
	Saturation OD	Doubling time (h)	Saturation OD	Doubling time (h)
YL	0.63 ± 0.01	4.7 ± 1.3	N/A	N/A
PK	1.06 ± 0.04	3.1 ± 0.09	0.58 ± 0.004	5.6 ± 0.29
V1	1.3 ± 0.04	3.7 ± 0.4	2.9 ± 0.09	2.8 ± 0.3

Y. lipolytica (YL), Vinasse isolated Pichia kudriavzevii strain (V1) and commercial Pichia kudriavzevii (PK) were grown on freshly diluted 1:1 vinasse. YL did not grow at 37 **º**C, which is denoted as N/A.

However, *P. kudriavzevii* strains were clearly more efficient than *Y. lipolytica* at 28 ºC. In addition, while *Y. lipolytica* did not grow at 37 ºC on vinasse (noted by N/A in table), both *P. kudriavzevii* strains were still able to grow slowly, demonstrating their natural capabilities to accommodate more stringent media and temperatures. When comparing directly *P. kudriavzevii* strains, while both strains had similar doubling time and saturation OD at 28 ºC, V1’s growth at 37 ºC was significantly more efficient than commercially available PK. This suggests that our isolated V1 strain was naturally more amenable to growth on vinasse and a potential strain to explore further for the production of lipids and vinasse valorisation.

### V1 *P. kudriavzevii* is naturally adapted for growth at higher temperature

**Fig. 1:**
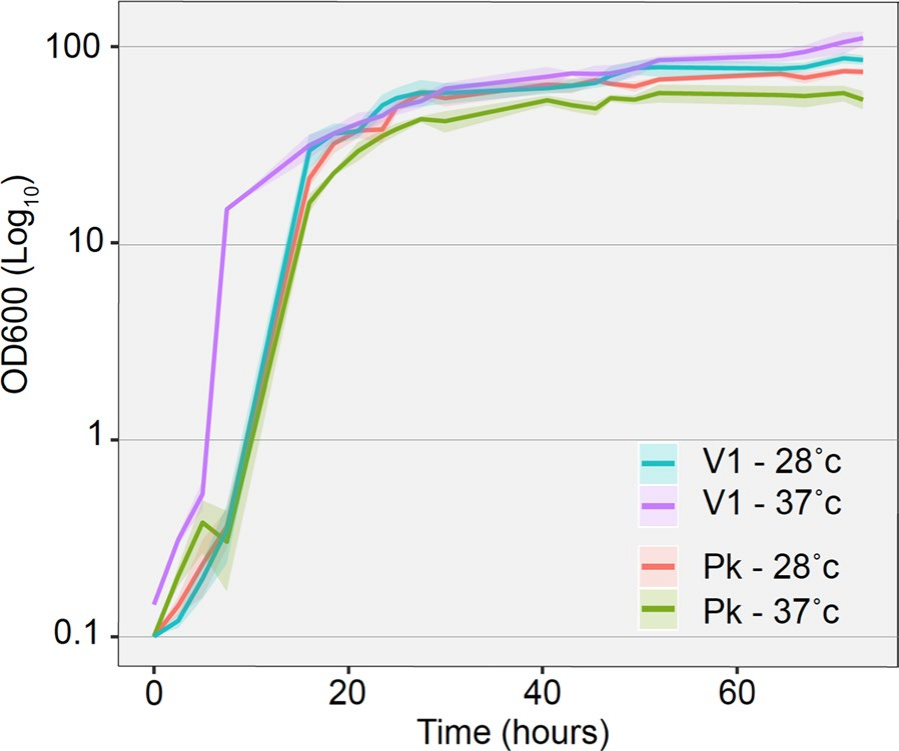
Liquid growth curve of yeast strains on YPV at either 28 ℃ or 37 ℃. V1 is *Pichia kudriavzevii* isolated from vinasse, and PK is commercial *Pichia kudriavzevii*

Temperature resistance is an important factor for efficient growth of yeasts on agricultural waste, since temperatures above 35 ℃ are expected in rum distilling countries. To investigate these results further, both *P. kudriavzevii* strains (commercial and V1*)* were grown at 28 ℃ or 37 ℃ on liquid YPV, to mimic vinasse growth (Fig. 1). The results suggest that while at 28 ℃, growth efficiency was comparable between PK strains, increasing the temperature to 37 ℃ resulted in slight decrease of commercial PK’s growth, while V1’s growth was improved at the higher temperature. Indeed, at 37℃, V1 doubling time is half that of 28 ℃ on both YPD and YPV (Table 4).

**Table 4:** Comparison of liquid growth on YPD and YPV at 28 ºC and 37 ºC

	YPD		YPV	
Yeast Strain	Saturation OD	Doubling time (h)	Saturation OD	Doubling time (h)
PK 28ºC	47.6 ± 2.05	2.12 ± 0.22	77 ± 0.47	4.06 ± 1.25
PK 37ºC	41.6 ± 0.47	1.6 ± 0.24	57 ± 2.49	6.13 ± 2.67
V1 28ºC	49.6 ± 1.2	2.03 ± 0.18	87 ± 4.3	3.70 ± 1.20
V1 37ºC	45.6 ± 3.3	1.16 ± 0.07	110 ± 7.4	1.54 ± 0.03

Vinasse isolated *P. kudriavzevii* strain (V1) growth was compared to commercial *P. kudriavzevii* (PK) for growth on glucose-based (YPD) and glycerol-based (YPV) media at 28 ºC or 37 ºC

The V1 strain also saturates at a higher OD at 37 ℃ than 28 ℃ (110 ± 7.4 compared to 87 ± 4.3) and higher in YPV than YPD at same temperature (87 ± 4.3 compared to 49.6 ± 1.2 for 28 ℃, respectively, and 110 ± 7.4 compared to 45.6 ± 3.3 for 37℃, respectively). In YPD, when glucose is the main carbon source, no significant growth difference was observed between strains at any temperatures (Table 4). While the saturation OD was slightly lower for both strains at 37 ℃ compared to 28 ℃, their doubling time were significantly shorter at 37 ℃ compared to 28 ℃ (Table 4). This suggests that V1 is naturally adapted for growth on glycerol-based carbon sources such as vinasse and can tolerate lower pH and higher temperatures.

**Fig. 2:**
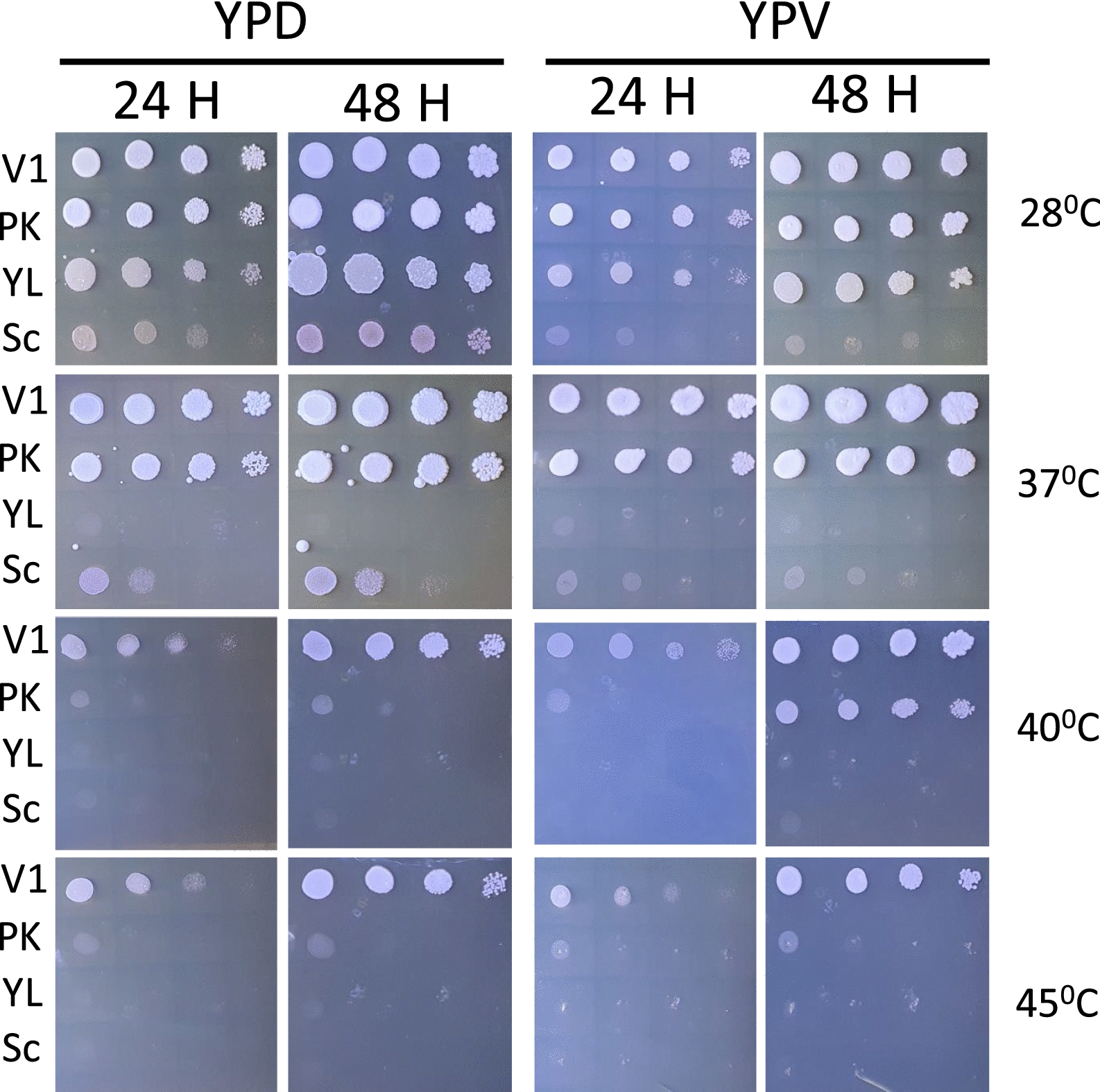
Temperature resistance of yeast strains. All yeast strains were grown at 28 ℃ in YPD media then serial diluted and spotted on either YPD or YPV media and incubated for 24 h and 48 h at various temperatures. V1 strain is the only strain growing at temperature above 40 ℃. V1: *Pichia kudriavzevii* from this study PK: commercial *Pichia kudriavzevii, YL: Yarrowia lipolytica,* Sc: *Saccharomyces cerevisiae*

To test V1’s resistance to a range of higher temperatures above 28℃, four strains (*S. cerevisiae*, *Y. lipolytica*, *P. kudriavzevii* and V1* P. kudriavzevii)* were grown at temperatures ranging from 28℃ to 45℃ on YPV. Figure 2 shows that all strains grew efficiently at 28℃ in YPD and YPV except for *S. cerevisiae* whose growth was reduced in YPV as expected, due to high glycerol content and inability to metabolise glycerol efficiently. At 37℃, the growth of both *S. cerevisiae* and *Y. lipolytica* were notably affected on both YPD and YPV. Under these conditions, both *P. kudriavzevii* strains still grew efficiently, with V1 being more efficient on YPV than the commercial PK, in agreement with previous results (Fig. 1 and Table 4). At 40℃, the growth of commercial *P. kudriavzevii* (PK) decreased dramatically, while V1 was still growing in both YPD and YPV. Finally, at 45℃, the commercial *P. kudriavzevii* stopped growing. In contrast, V1 continued to grow on both media, although at a slower rate than 28 ℃. Above 50 ℃ none of the yeasts showed any growth (not shown). These results demonstrate that the strain V1 *P. kudriavzevii* isolated from vinasse is the best growing yeast strain in our hands, with sustained growth up to 45℃ at high glycerol content.

### V1 *Pichia kudriavzevii* is resistant to low pH

**Fig. 3:**
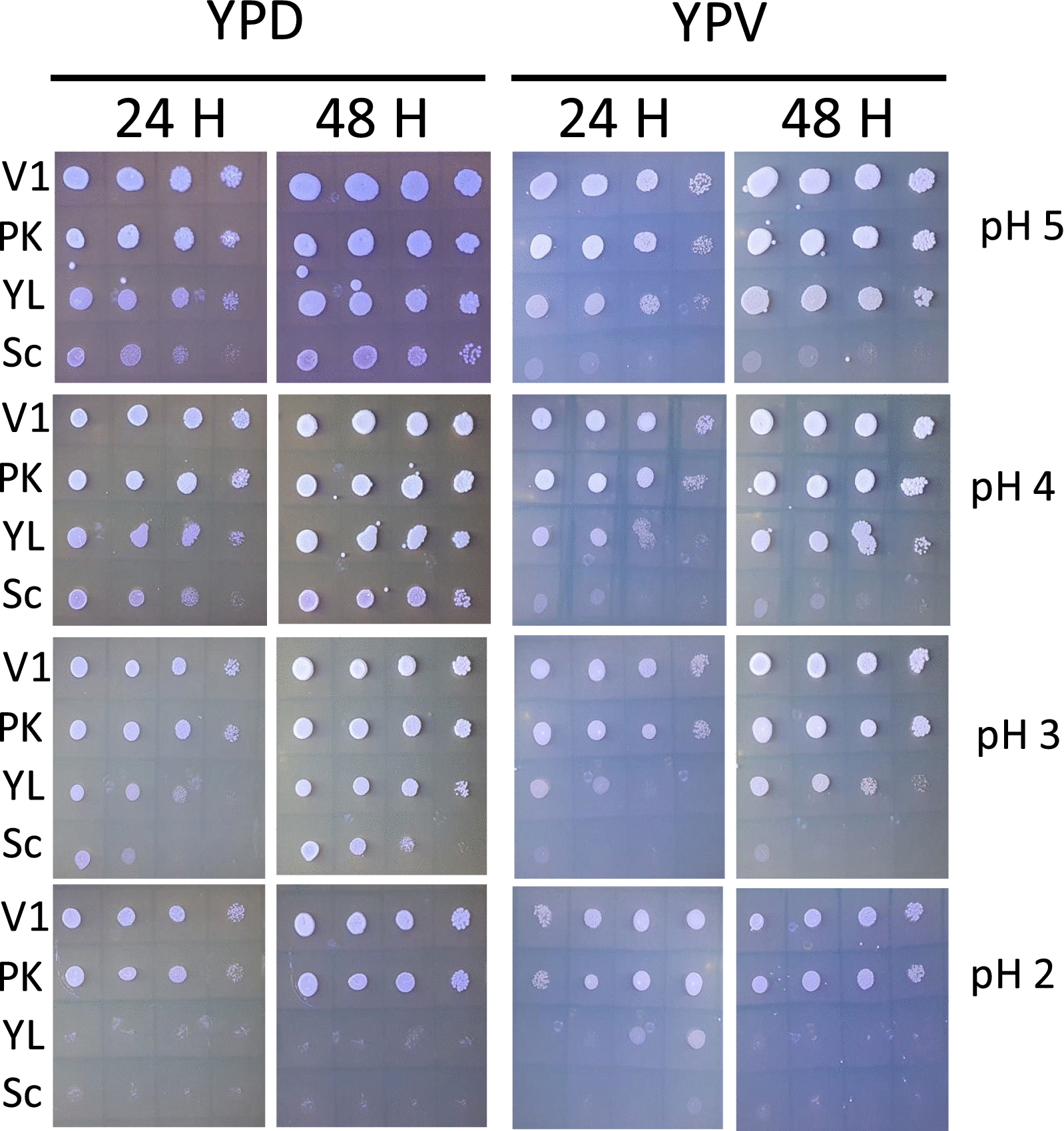
V1 is resistant to acidic pH. All yeast strains were grown at 28 ℃ in YPD then serial diluted and spotted on either YPD or YPV media and incubated for 24 h and 48 h at various pH. V1: *Pichia kudriavzevii* from this study PK: commercial *Pichia kudriavzevii, YL: Yarrowia lipolytica,* Sc: *Saccharomyces cerevisiae*

Agricultural waste such as vinasse, mango or cashew apple fruit juice are usually characterised by an acidic pH [21, 54, 55]. Resistance to acidic pH is, therefore, one of the important factors for evaluating the suitability of yeast strains to grow in vinasse and other agricultural waste. We tested growth of V1 on both YPD and YPV at pH ranging from 5 to 2 and compared to the growth of control strains (Fig. 3). As expected, all five strains grew efficiently at pH 5 on both YPD and YPV, apart from *S. cerevisiae* which could not grow on high glycerol content media as expected.

The growth of *Y. lipolytica* started to decrease at pH 3 and no growth was observed at pH 2 for this strain, independent of the media. Yet, both *P. kudriavzevii* strains were able to maintain growth at pH 2, albeit less efficiently compared to pH 5. No difference could be seen between YPD and YPV for both *P. kudriavzevvii* strains at pH 2.

This demonstrates that *P. kudriavzevvii* strains are very tolerant to acidic pH, which suggests that these strains are well-adapted for growth on agricultural waste, such as vinasse. In particular, V1 is also additionally naturally very well tolerant to higher temperature climates (between 37 ℃ and 45 ℃) (Figs. 1 and 2) and, therefore, represents a very good yeast candidate for lipid production in these conditions.

### V1’s potential for bio-engineering

With encouraging results on temperature and pH resistance, the potential of V1 to be genetically engineered was tested. Since *P. kudriavzevii* does not yet have a toolbox of vectors, we explored conventional selection techniques using either auxotrophic markers or antibiotic markers.

**Fig. 4:**
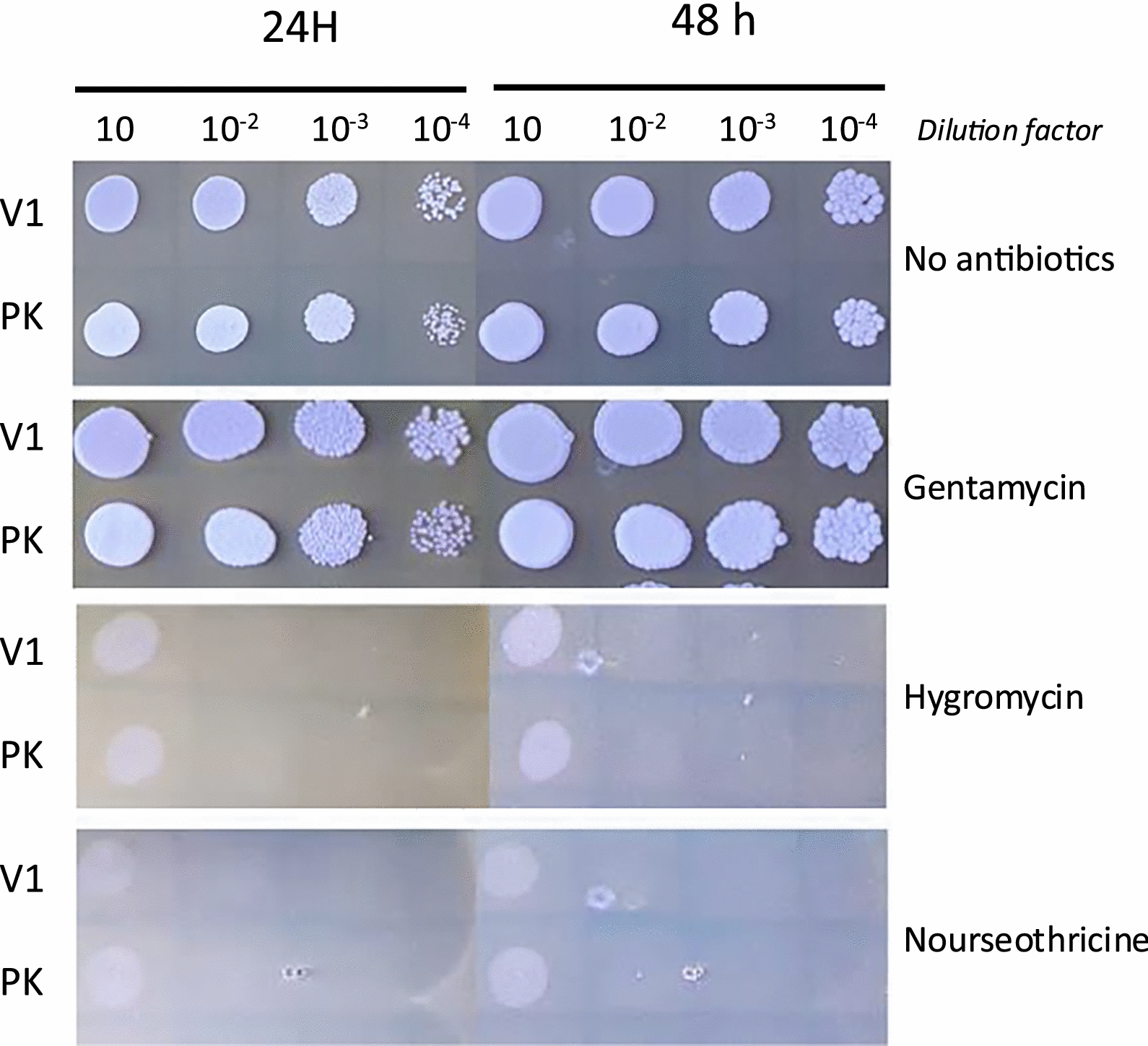
V1 is sensitive to hygromycin and Nourseothricine. Various dilution of V1 and PK were spotted on YPD containing various antibiotics (300ug/ml) and growth was observed after 24 h or 48 h at 28℃. V1: *Pichia kudriavzevii* from this study PK: commercial *Pichia kudriavzevii.* Dilution of yeast culture before plating is mentioned

We first tested the V1 strain’s ability to grow on minimal media and in the presence of antibiotics. Predictably, V1 was prototrophic to all amino acids classically used as a selection for *S. cerevisiae,* such as tryptophan, uracil, histidine, leucine or adenine (data not shown). Therefore, classical cloning in plasmids with auxotrophic markers was not pursued. Instead, antibiotic resistance, such as gentamicin, hygromycin and nourseothricin, commonly used as a selection to transform *Y. lipolytica,* was tested [43]. As shown in Fig. 4, both *P. kudriavzevvii* strains, V1 and PK, are resistant to gentamicin but sensitive to hygromycin and nourseothricine. We, therefore, tested if V1 could be transformed by classical heat shock transformation with a vector using one of these antibiotics as the selection marker.

**Fig. 5:**
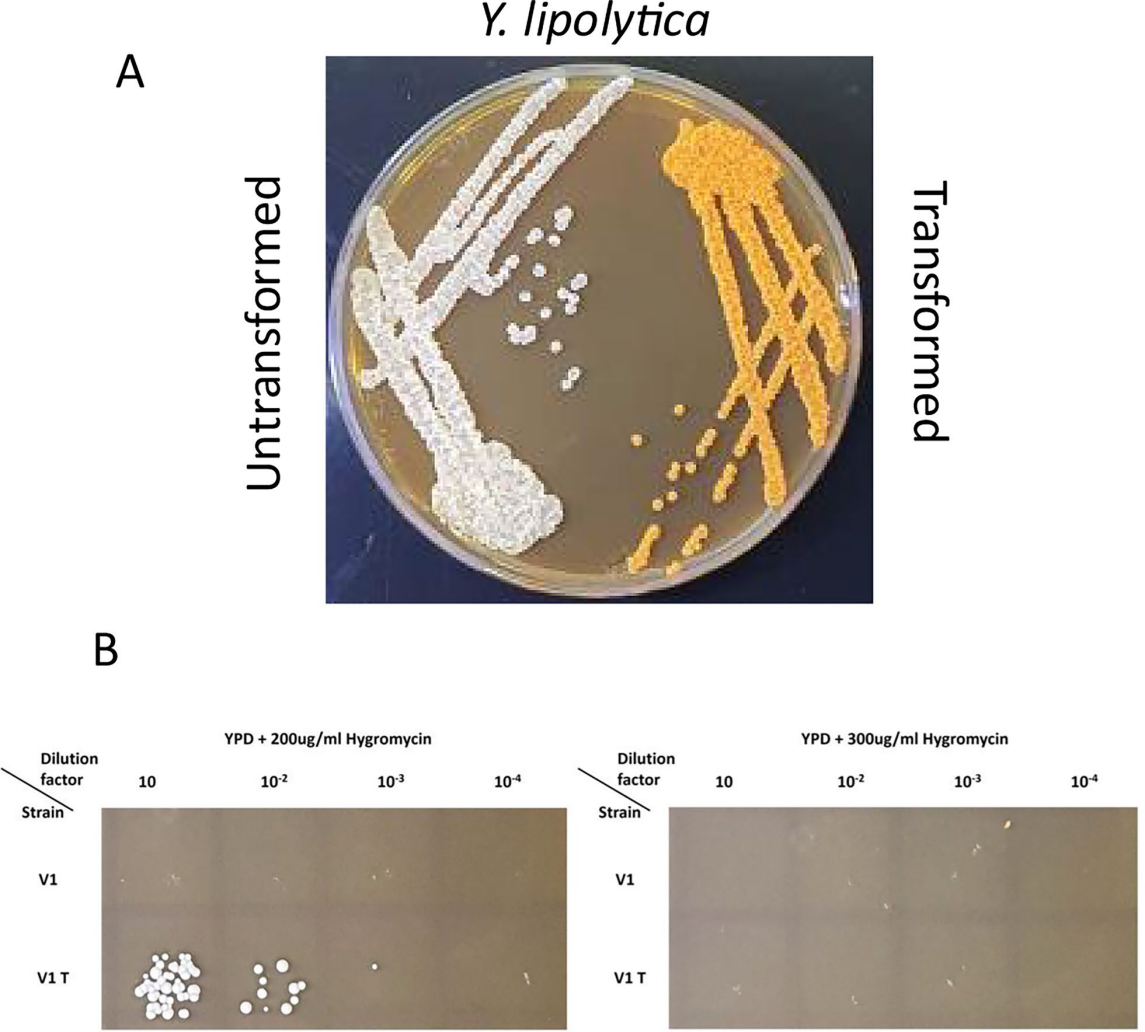
Yeast transformation results with *Y. lipolytica* plasmid. **A** Wild-type *Y. lipolytica* was transformed with a plasmid containing a cassette of genes for the production of β-carotene [28] and selected on YPD with hygromycin. Colonies were then streaked onto YPD without antibiotic next to an untransformed control to show the colour change. **B** Growth of V1 (*P. kudriavzevii* isolated from vinasse) untransformed (V1) or transformed with the same *Y. lipolytica* plasmid (V1T) as in (**A**), and selected on two concentrations of hygromycin, showing V1T slow growth at lower hygromycin concentration but without colour change

To this purpose, a vector commonly used to transform *Y. lipolytica*, selectable on hygromycin and containing a cassette of genes for the production of β-carotene, was used to transform V1. The production of β-carotene leads to orange colonies when efficiently expressed in *Y. lipolytica* and will, therefore, indicate if this gene could also be translated in V1 [28]. As a positive control, *Y. lipolytica* was transformed with the same vector, which lead to the production of β-carotene causing the expected colour change for transformed colonies (Fig. 5A). Upon transformation of *P. kudriavzevii* with the *Y. lipolytica* plasmid, no growth was observed at 300 ug/ml hygromycin (Fig. 5B, right panel). However, when the concentration was reduced to 200 µg/ml hygromycin, a slight growth was observed for the transformed strain, while the untransformed remained sensitive to hygromycin at the same concentration (Fig. 5B, left panel). This suggests that while the hygromycin resistance gene contained in the plasmid was not adapted for *P. kudriavzevvii*, it was still partially functional in this strain to allow resistance at 200 µg/ml hygromycin. The lack of orange colour development, however, could indicate that the *Y. lipolytica* genes did not express in these conditions, or that the promoter or the enzyme was not functional in *P. kudriavzevvii*. Nevertheless, this result demonstrates that *P. kudriavzevii* can be transformed by the classical heat shock protocol. Although the YL genes might not be functional in *P. kudriavzevii*, partial growth on low concentrations of hygromycin show that transformation was efficient and the kinase conferring resistance to hygromycin was partially functional. While further optimisation would be necessary, these results strongly support that V1 has the potential to be engineered by classical methods.

### Whole genome sequencing of V1

**Fig. 6:**
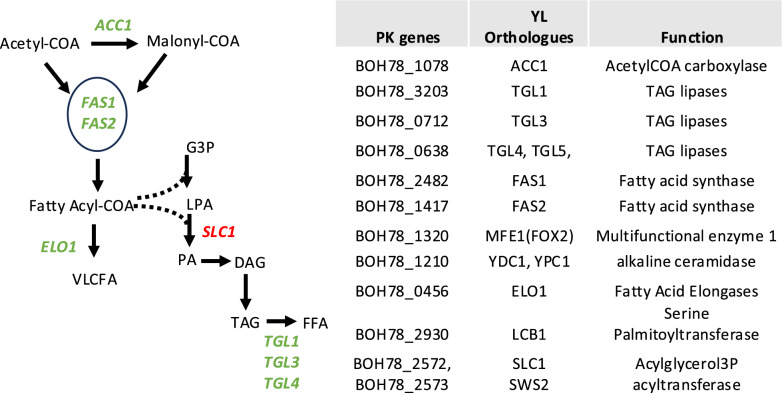
GO enrichment in genes involved in V1 lipid metabolism. The figure and table showing the enzymes from V1 containing high levels of SNPs involved in the free fatty acids (FFA) synthesis pathway. SNPs are enriched in genes in green. The lipid metabolism of P. kudriavzevii is not known; therefore, the left panel is adapted from the lipid biosynthesis pathway in *Y. lipolytica* (adapted from Bredeweg et al. [56]). WGS can be found in supplemental Table S2

To gain a better understanding of the genome of V1 and most importantly genes naturally present involved in the metabolism of lipids, which will be important for biofuel production, a whole genome sequencing analysis was performed and compared with established genomes of *P. kudriavzevii* strain. Analysis of single-nucleotide polymorphisms (SNPs) was performed (Table S2). GO enrichment analysis of non-synonymous SNPs related to lipid metabolism revealed a significant enrichment in genes encoding for enzymes involved in the synthesis of lipids. In particular, a number of tri-acylglycerol (TAG) lipases were present in this selection and represented in Fig. 6. Since the lipid metabolism pathway of V1 is not known, the SNPs from genes associated with lipid metabolism are represented from the *Y. lipolytica* pathway [56]. In many oleaginous yeasts, lipids are stored mainly as TAGs [57]. Intracellular lipases such as the triacylglycerol lipases TGL3 and TGL4 were shown to be involved in TAG remobilisation, by degrading TAGs into free fatty acids (FFAs). Inactivation of these lipases in *Y. lipolytica* have led to higher levels of lipid accumulation and it would be interesting to test if inactivation of the V1 orthologues of TGL3 and TGL4 could improve FFA quantities [58]. In the meantime, enrichment of SNPs in genes involved in lipid metabolism suggests that this strain possesses lipid metabolism pathway(s) adapted to its stringent growth conditions which merits further exploration.

### Lipidomic comparison of *P. kudriavzevii *V1 and *Y. lipolytica* YL

To understand how the lipid profile of with V1 compares to the well-characterised lipid profile of the oleaginous *Y. lipolytica* and to evaluate which types of lipids V1 could produce that would be useful in biofuel production, a lipid analysis was performed. Both strains were grown in YPV at 28 °C. In addition, since V1 appeared to grow more efficiently at 37 °C, we also compared the lipid profiles of V1 grown at 28 °C and 37 °C, to gain further understanding in lipid metabolism that might provide insight into the thermo-resistance of V1.

**Fig. 7:**
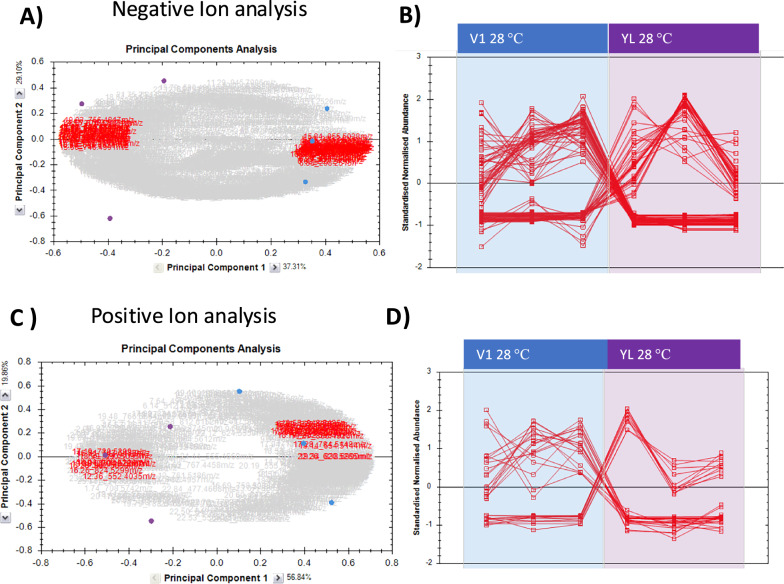
Principal component analysis and distribution of normalised abundances of features in V1 samples compared to *Y. lipolytica* samples*,* both grown at 28 °C. **A**, **B** Progenesis analysis from negative ion electrospray data, while (**C**) and (**D**) show the analysis from positive ion electrospray data. **A**, **C** PCA plots showing separation of the data sets; the triplicates for V1 at 28 °C are blue circles and triplicates for YL at 28 °C are purple circles. All the features are indicated by the grey cloud and features with power > 0.95 in red. **B**, **D** Distribution of normalized abundance between the 3 replicates in each of the two data sets (triplicates for V1 28 °C in the blue box and triplicates for YL 28 °C in the purple box)

**Fig. 8:**
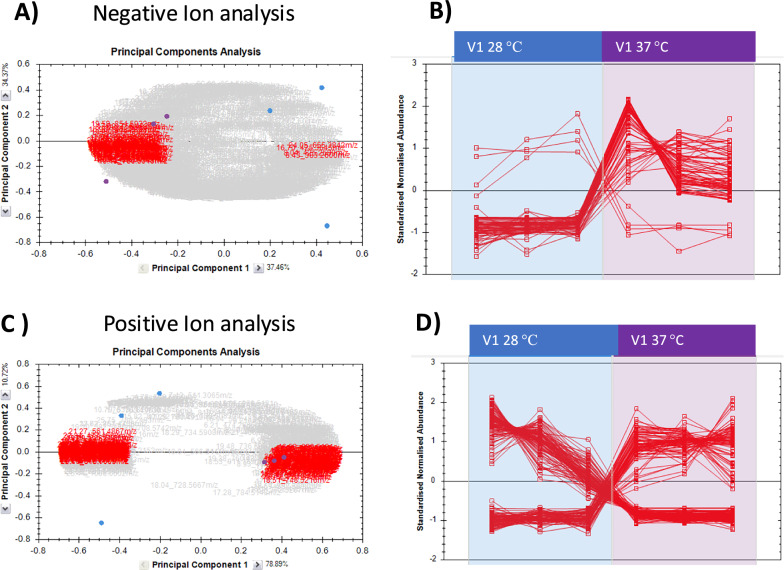
Principal component analysis and distribution of normalised abundances of features in V1 samples grown at 28 °C compared to 37 °C. **A**, **B** Progenesis analysis from negative ion electrospray data, while (**C**) and (**D**) show the analysis from positive ion electrospray data. **A**, **C** PCA plots showing separation of the data sets; the triplicates for V1 at 28 °C are blue circles and triplicates for V1 at 37 °C are purple circles. All the features are indicated by the grey cloud and features with power > 0.95 in red. **B**, **D** Distribution of normalized abundance between the 3 replicates in each of the two data sets (triplicates for V1 28 °C in the blue box and triplicates for V1 37 °C in the purple box)

**Fig. 9:**
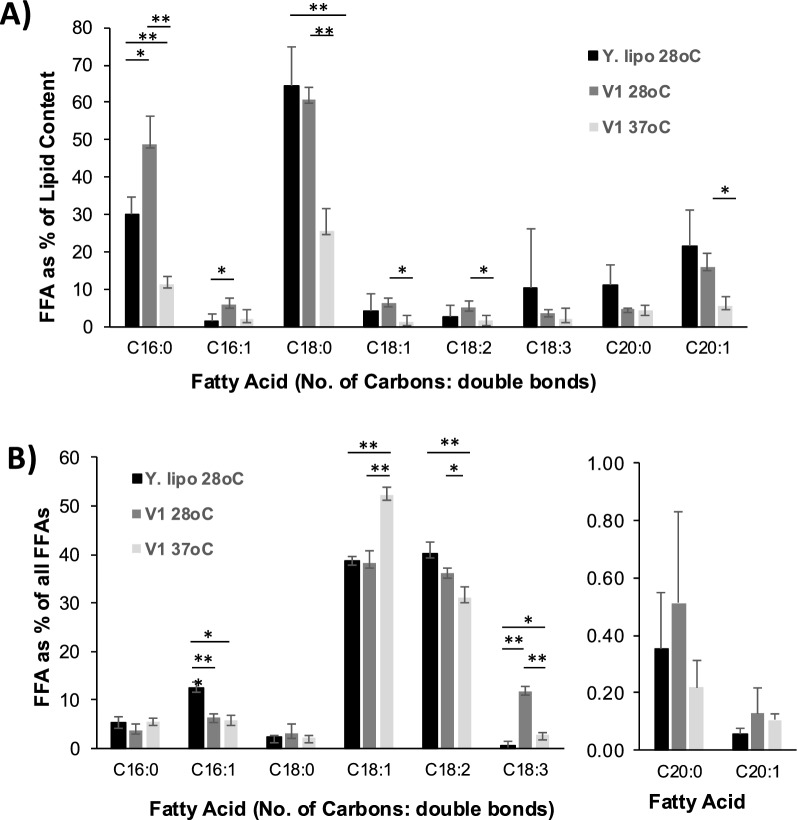
Relative quantification of free fatty acid (FFA) content in YL, V1 28 ℃, V1 37 ℃. **A** Each free fatty acid calculated as a percentage of all the lipid classes (FFAs, Lysolipids, phospholipids and triacylglycerols) containing that specific fatty acyl chain, determined from the MS^2^ extracted ion chromatogram in negative ion mode. **B** Each free fatty acid calculated as a percentage of all the FFAs analysed in each sample, determined from the data used to plot (**A**). For statistical analysis *p* < 0.01 are represented by two stars. *p* < 0.05 are represented by one star

As expected, both the *Y. lipolytica* and *P. kudriavzevii* lipid profiles contained a wide variety of lipids commonly found in yeast including free fatty acids (FFAs), ceramides, phosphatidylcholines (PC), phosphatidylethanolamines (PE), phosphatidylserines (PS), phosphatidylinositols (PI), triacylglycerides (TAG) and phosphatidylglycerols (PG) [59, 60]. Although there was some variability within the biological replicate within each set, these were minor compared to the differences between the strains or within the same strain grown at different temperatures, where the lipidomic profiles differed significantly (Figs. 7, 8 and 9). It should be noted that the tandem MS analysis was carried out using data-independent analysis (MS^e^), which makes it harder the relate the fragment ions to the parent ion. Therefore, while it was possible to determine the sum composition of the fatty acyl chains in the lipids, it was challenging to assign specific fatty acyl chains to individual features. Consequently, the most likely positional isomers for the features are given in the supplementary Tables S3 and S4.

For YL and V1 strain grown at 28 °C, the principal component analysis (PCA) of data acquired in both negative (Fig. 7A, B) and positive ion electrospray mode (Fig. 7C, D) shows a clear separation between V1 and YL. Features contributing to the separation are listed in Table S3. Some general trends were apparent from this analysis; for example, V1 tended to have higher levels of more PCs with highly unsaturated acyl chains, while YL tended to contain higher levels of certain PE and PS species with shorter acyl chains and relatively few double bonds. Examination of the lipids that contributed the most to the separation in the PCA plot also identified increased levels of some specific lysolipids (LPC) and free fatty acids, and a short chain PI with acyl chain sum composition C28:1 was highly enriched in V1 compared to YL. On the other hand, several PE, PS and PI species had lower levels in V1 compared to YL.

When V1 lipid profiles from growth at 28 °C and 37 °C were compared, PCA analysis of the positive and negative ion data again showed clear separation in both negative ion mode (Fig. 8A) and positive ion mode (Fig. 8C). Most strikingly, in the negative ion analysis, many more features increased at the higher temperature than at lower temperature (Fig. 8B and Table S4), whereas in positive ion mode a similar number of features increased and decreased at 37 °C. The features contributing most to the separation are listed in Table S4. Overall, lipid species that increased most in V1 grown at 37 °C were mainly PC species: more than 12 different PC species were increased and several of those showed very high max-fold changes (> 25-fold), although a few PE and PI species also increased. In contrast, the species that increased significantly in V1 samples grown at 28 °C showed relatively smaller max-fold changes (< 20-fold). While the largest increases in abundance at 28 °C were a PI and a PE species, it was interesting that 5 ceramides and 2 cardiolipins had higher levels, as well as some shorter chain TGs and a DG.

To analyse further the differences in fatty acid composition between the samples, extracted ion chromatograms (XIC) were generated from the negative ion MS^e^ data for a range of expected fatty acid fragment masses (examples for lipids generating palmitoleoyl and oleoyl fragments are shown in Figs. S1, 2, 4 and 5). Chromatograms were integrated using the MassLynx software. This provides a relative quantitative profile of the abundance of these fatty acid chains in phospholipids and glycerolipids that are ionisable in negative ion mode. Significant differences in fatty acid profiles were also observable between the strains and reinforced the previous analysis (Fig. 9). A similar approach identified phosphocholine-containing lipids (PCs) by generating XICs for the diagnostic fragment ion at m/z 184.07 (Figs. S3 and S6).

In particular, relative quantitative analysis of the XIC data for specific FFAs shows higher levels of C16:0 and C16:1 in V1 28 °C compared to YL with similar levels for other lipid species (Fig. 9A). In addition, V1 grown at 28 °C had significantly higher levels of lysolipids and FFAs compared to YL (Figs. S1–S3), and slightly higher levels of earlier eluting phospholipids species were also observed at retention times < 17 min. This suggests that V1 28 °C accumulates shorter or more unsaturated fatty acids compared to YL. In particular, C18:3 is significantly more highly represented amongst total FFA in V1 28 °C compared to YL (Fig. 9B).

Since growth of V1 at 37 °C was more efficient than at 28 °C (Fig. 2 and Table 3), V1 37 °C was compared to V1 28 °C following the same analysis as above. V1 37 °C generally had overall lower (C16:0, C16:1, C18:0, C18:1, C18:2, and C20:1) or similar (C18:3 and C20:0) amounts of FFAs as a proportion of total lipids to V1 28 °C (Fig. 9A), suggesting that V1 37 °C accumulates other types of lipids rather than FFAs. Within FFAs, V1 37 °C specifically accumulated more oleic acid (C18:1) compared to both V1 28 °C and YL (Fig. 9B and S5), and less linolenic acid (C18:3) than V1 28 °C (Fig. 9B). In addition, no obvious changes in the amount of lysolipids were observed compared to V1 28 °C (Figs. S4–S6). However, V1 37 °C showed slightly lower levels of earlier eluting phosphatidylcholine species (Rt < 17 min) and more containing longer or more saturated acyl chains, eluting a later in the chromatogram (21–22 min) compared to V1 at 28 °C, supporting a shift to longer chains or reduced unsaturation, consistent with adapting to the increased fluidity resulting from the switch of temperature to 37 °C (Fig. S6).

## Conclusions and discussion

Agricultural waste products, such as vinasse, are an escalating issue in developing countries, posing significant environmental challenges. Often left to decompose on fields, they contribute to sanitation problems and environmental toxicity. With the urgency to find sustainable disposal solutions for agricultural waste, this study addresses this critical concern by exploring the potential of vinasse, a byproduct of rum distilleries, as a medium for biofuel production. Vinasse is notoriously harsh, containing high glycerol levels, low pH, and a variety of ions and metals. Its composition varies considerably, making it difficult to identify yeast strains capable of thriving in such conditions.

Our research focused on identifying yeast strains that could grow efficiently on vinasse. While attempts to engineer classical strains such as *S. cerevisiae* and the oleaginous *Y. lipolytica* showed limited success, we identified a promising new strain, *P. kudriavzevii* (V1), which demonstrated robust performance in vinasse-like conditions. This strain exhibited exceptional resistance to both higher temperatures (up to 45 °C) and acidic pH (as low as pH 2), significantly outperforming commercial strains. V1 outperformed other strains in extreme growth conditions, demonstrating greater flexibility and a potential for optimization in industrial applications.

To test this, we successfully transformed V1 using a standard lithium heat shock protocol, introducing a plasmid from *Y. lipolytica*. While the transformation efficiency was modest, this breakthrough suggests that V1 has substantial potential for genetic engineering to enhance its growth on vinasse.

The presence of significant SNPs in genes related to lipid metabolism further supports the idea that these genes are involved in V1 survival in vinasse and that the strain could be used for biofuel production based on its fatty acids. Indeed, many yeasts, including *S. cerevisiae* and *Y. lipolytica*, adjust their lipid composition upon stresses, such as temperature, glycerol content and pH [59, 61–63]. Therefore, this could represent a coping strategy for growth under extreme conditions, such as vinasse.

In addition, the SNPs analysis also suggested that V1 could be a good biofactory for lipid production.

This was confirmed by the lipidomics analysis which revealed key insights into the strain's potential for biofuel production. At 28 °C, V1 accumulated higher levels of specific FFA, such as palmitic and palmitoleic acid (C16:0 and C16:1). In contrast, V1 grown at 37 °C showed decreased amounts of overall FFAs and no changes in lysolipids, suggesting that other types of lipids, for example, triacylglycerols, could be enriched at 37 °C. Indeed, several TAG lipases were identified with enriched SNPs from the WGS (Fig. 6) which would support this hypothesis. In addition, given that 37 °C (or higher) is the usual growth temperature in vinasse-producing countries (in contrast to the laboratory temperature strains grown at 28 °C), these findings would suggest that V1 naturally produces a lipid profile that could be ideal for biofuel production. Our results additionally show a shift to longer chains or less unsaturation, with an enrichment in oleic acid (C18:1) amongst other FFAs. This is in agreement with a previous study showing that *P. kudriavzevii* isolated from rotten fruits contained high levels of oleic acid followed by palmitic, linoleic acid, and stearic acid and was suitable for biofuel production [48]. Indeed, the nature of fatty acids is critical for the quality of biofuel. Palmitic acid (16:0), stearic acid (C18:0), oleic acid (C18:1), linoleic acid (C18:2), and linolenic acid (C18:3) have been described as most important for biofuel quality [64]. TAG and FFAs are crucial lipid types for the production of biofuels [65]. Therefore, this suggests that V1 *P. kudriavzevii* naturally produces the crucial set of important lipids which could have the potential to be engineered for the optimisation of biofuel production.

While *Y. lipolytica* has been the most popular and successful strain for lipid production, recent studies increasingly highlight the advantages of *P. kudriavzevii* from agricultural waste, further validating our approach [54, 66–68]. In addition, CRISPR technology has been recently applied to engineer this strain for organic acid production, complementing our findings and underscoring the strain's industrial potential [69].

At this stage, more research is required to characterise the growth of this newly identified strain V1 on vinasse media, and help the rum industry become more sustainable [70]. Most importantly, a further in depth analysis of lipidomics on freshly produced vinasse will help establish whether this strain would naturally produce enough fatty acids for biofuel production, or if further engineering is needed. This study has shown the feasibility of the two approaches that can be investigated in parallel to evaluate the full potential of *P. kudriavzevii* (V1). The results so far suggest that this newly identified *P. kudriavzevii* (V1) strain is uniquely adapted to thrive on vinasse and at temperatures between 37 °C and 45 °C normally found in rum distilling countries, positioning V1 as a strong candidate for future biofuel production and agricultural waste valorisation. This study opens the door for industrial bioengineering innovations, paving the way for sustainable solutions in the agricultural sector and the rum industry.

## Supplementary Information

Supplementary Material 1.

Supplementary Material 2.

Supplementary Material 3.

Supplementary Material 4.

## Data Availability

The Whole genome sequencing is available publicly.The raw V1 reads for this study have been deposited to NCBI GenBank BioProject accession number PRJNA1254219 and the SRA database with the accession SRX28499329. The microbiome, SNPs analysis and lipidomics raw data are provided as supplemental data in this submission. The raw data from presented analysis is available upon request to the senior authors.

## Abbreviations

YPD: Yeast peptone dextrose
YPV: Yeast peptone vinasse
SC: *Saccharomyces cerevisiase*
PK: *Pichia kudriavzevii*
YL: *Yarrowia lipolytica*
OD: Optical density
CRISPR: Clustered Regularly Interspaced Short Palindromic Repeats
WGS: Whole genome sequencing
SNPs: Single-nucleotide polymorphisms
PCA: Principal component analysis
FFA: Free fatty acids
PC: Phosphatidylcholine
PE: Phosphatidylethanolamines
PS: Phosphatidylserines
PI: Phosphatidylinositols
TAG: Triacylglycerides
PG: Phosphatidylglycerols
DG: Diacylglycerols
