# Supplementary material for: A new yeast strain for valorisation of vinasse, a rum distillery waste product
Source: Biotechnol Biofuels Bioprod. 2025 Jul 18;18:76. doi: 10.1186/s13068-025-02671-0 (PMC12273314; doi:10.1186/s13068-025-02671-0)
Supplement: Supplementary file 1 — Supplementary Material 1. [file 13068_2025_2671_MOESM1_ESM.pdf]

Eurofins Genomics GmbH, Anzinger Str. 7a, D-85560 Ebersberg

Data Analysis Report

Project / Study: GEN180710_P

Project specification: Microbiome Profiling

Date: September 10, 2018

Table of Contents

1 Microbiome Analysis Pipeline **1**

2 FASTQ Read Statistics **3**

3 Read Merging **4**

4 Microbiome Profiling **5**

4.1 Results 5

4.1.1 Statistics 5

4.1.2 Taxonomic Composition of Samples 6

4.2 Methods 10

4.3 Output Files and Descriptions 11

1 Microbiome Analysis Pipeline

The microbiome analysis pipeline consists of three major steps and some intermediate filtering steps. Each major pipeline step is described in more detail in its respective report section. The following list provides an overview of the full pipeline, while the **main results** of the microbiome analysis are presented in section *Microbiome Profiling*.

Demultiplexing All reads passing the standard Illumina chastity filter (PF reads) are demultiplexed according to their index sequences.

Primer clipping The target region specific forward and reverse primer sequences are identified and clipped from the starts of the raw forward and reverse reads. If primer sequences could not be perfectly matched (no mismatches allowed), read pairs are removed at this step to retain only high-quality reads. The information on the remaining read pairs are provided in section *FASTQ Read Statistics*. The files with clipped reads are provided in the FASTQ directory and are named `*_1.fastq.gz` and `*_2.fastq.gz`. These files are not directly used as inputs for the final microbiome profiling, but are further processed as described in the following steps.

Merging If the ends of forward and reverse reads overlap, the reads are merged (assembled) to obtain a single, longer read that covers the full target region. If the target region is longer than two times the read length, merging should be impossible. If in such a case a read pair can still be merged, it is considered as an artifact and will be removed in the following quality filtering step. If the target region is only slightly shorter than two times the read length, merging my fail due to an insufficiently long high-quality overlap of the read ends. In such a case, typically only a fraction of the read pairs can be merged. In all abovementioned cases where some read pairs can't be merged, the forward read is retained and processed in the following steps instead.
In short, reads are merged if possible, and as a fallback the high quality forward read is used. No read pair is completely discarded in this step. See section *Read Merging* for additional details.

Quality filtering Merged reads are length filtered according to the expected length and known length variations of the target region (see table 1). Merged reads that are significantly shorter than the expected minimal target region length, or that are significantly longer than the expected maximal target region length, are discarded at this step. The ends of retained (not-merged) forward reads are clipped to a total read length of 276 bp to remove low quality bases. Merged and retained reads containing ambiguous bases ("N") are discarded.
The files with filtered reads are provided in the FASTQ directory and are named `*.merged_for_profiling.fastq.gz`. These files are used as inputs for the following microbiome profiling.

Microbiome profiling The length filtered merged reads and the quality clipped retained forward reads are used as input for the microbiome profiling, where as a first step chimeric reads are identified and removed. All details of the microbiome step can be found in section *Microbiome Profiling*:

- Methods description of chimera removal, OTU picking, taxonomic assignment, etc.
- Tables with statistics describing the results of microbiome profiling
- Overview of the taxonomic composition of samples
- Detailed descriptions of delivered result files

© Eurofins Genomics

Region code	Expected length	Merging efficiency
16S	ca. 395 bp	high
A16S	ca. 645 bp	not expected
COI	ca. 650 bp	not expected
CYTB	(highly variable)	(highly variable)
FUNGIF1	ca. 290 bp	high
ITS1	(highly variable)	high
ITSA	ca. 445 bp	high
ITSB	ca. 350 bp	high
STAATS	(highly variable)	high
V1V3	ca. 490 bp	moderate
V3V4	ca. 445 bp	high
V3V5	ca. 600 bp	not expected

Table 1: Standard target regions, expected lengths (rough average), and expected merging efficiency.

2 FASTQ Read Statistics

The processing of sequencing reads according to primer sequences has been performed with in-house scripts. Only read pairs where the expected forward primer as well as the expected reverse primer were found have been kept for further analysis. For the identification of primer sequences no mismatches were allowed. The following table provides various statistics describing the sorted reads.

Table 2: FASTQ processing results.

No	Sample	Read Pairs	Yield (Kbp)	%Q30	Mean Q
1	A1.ITS1	87,351	48,741	63.47	28.90
2	A1.ITS2	195,291	109,362	71.49	31.36
3	A2.V1V3	95,497	53,669	75.98	32.38
4	A3.V3V4	176,286	100,306	77.87	32.86
5	B1.ITS1	71,193	39,725	78.12	32.87
6	B2.V1V3	155,070	87,149	75.23	32.21
	**Total/Average**	**780,688**	**438,952**	**73.69**	**31.76**

Remarks:

- All reads are passed filter, i.e. reads have passed the default Illumina filter procedure (chastity filter).
- "Yield (Kbp)": number of bases called in kilobases.
- "%Q30": represents the percentage of bases with a quality score of at least 30 (inferred base call accuracy of 99.9%). The Q-score is a prediction of the probability of a wrong base call.

3 Read Merging

Paired-end reads were merged using the software FLASH (2.2.00, Magoc and Salzberg, 2011). Briefly, the FLASH algorithm considers all possible overlaps at or above a minimum length between the reads in a pair and chooses the overlap that results in the lowest proportion of mismatched bases in the overlapped region. FLASH computes a consensus sequence in the overlapped region by selecting at each overlapped position the base with the higher quality value. If both bases have an identical quality value, one is selected randomly. Pairs were merged with a minimum overlap size of 10bp to reduce false-positive merges.

Table 3: Results of read merging.

**Sample**	**Total pairs**	**Percent combined**	**Mean of lengths**
A1.ITS1	87,351	98.1%	148
A1.ITS2	195,291	88.1%	273
A2.V1V3	95,497	73.3%	477
A3.V3V4	176,286	91.2%	413
B1.ITS1	71,193	91.6%	379
B2.V1V3	155,070	84.3%	439

Citation:

- Magoc T and Salzberg S (2011) FLASH: Fast length adjustment of short reads to improve genome assemblies. Bioinformatics 27 (21), 2957-63

4 Microbiome Profiling

4.1 Results

This section summarizes the results of read preprocessing, OTU picking, and taxonomic assignment. A description of the applied methodology and according literature references are provided in the section *Methods*. Descriptions of result files and visualizations are provided in the section *Output Files and Descriptions*.

4.1.1 Statistics

Total number of input sequences	780,317	100.0%
Remaining sequences after preprocessing and quality filtering	780,014	100.0%
Remaining sequences after chimera detection and filtering	777,979	99.7%
Total number of sequences assigned to OTUs	622,439	79.8%
Total number of sequences assigned to taxa	620,101	79.5%
Copy-number corrected total count	490,475	-
Total number of OTUs	955	100.0%
Number of OTUs assigend to taxa	953	99.8%

Table 4: Summarized statistics

The number of OTUs correlates with the diversity of the data set. Sequences that were considered as noise by the OTU picking algorithm were not assigned to an OTU. The fraction of OTUs that could be assigned to taxa indicates how well the microbiome is represented in the used reference database. A copy-number correction was performed for bacterial species only, see Angly FE et al. (2014). To do so, the number of reads assigned to a species was divided by the known or assumed copy-number of marker genes/regions. The resulting corrected total count may be significantly lower than the (raw) total number of assigned reads.

**Sample**	**1)**	**2)**	**3)**	**4)**	**5)**	**6)**
A1.ITS1	87,197	99.7%	91.0%	88.8%	77,432	149
A1.ITS2	195,290	100.0%	81.1%	81.1%	158,289	276
A2.V1V3	95,484	100.0%	73.3%	72.9%	24,114	471
A3.V3V4	176,175	99.9%	75.8%	75.8%	49,323	421
B1.ITS1	71,102	100.0%	74.2%	74.2%	52,770	427
B2.V1V3	155,069	98.8%	82.9%	82.9%	128,547	438

Table 5: **1)** Input sequences. **2)** Sequences after preprocessing and chimera removal. **3)** Sequences assigned to OTUs. **4)** Sequences assigned to taxa. **5)** Count after lineage-specific copy-number correction. **6)** Median sequence length after preprocessing.

The tables can be found as files in the results directory. Please see the according section for details about result files.

4.1.2 Taxonomic Composition of Samples

The following table provides an overview of the identified taxonomic units in each sample. The most specific taxonomic units are listed with their taxonomy level and fraction (k...kingdom, p...phylum, c...class, o...order, f...family, g...genus, s...species). The most specific taxonomic unit is the lowest common taxonomic unit of the listed species (small font). These species came up as best hits of the OTUs representative sequences during the database comparison.
Next to each sample name, the corrected total number of reads of this sample that were assigned to OTUs is given. All taxonomic units with less than 0.0% of reads are collapsed in the category "Other". If the representative sequence of an OTU had no significant database match, no taxonomic unit could be assigned. The total number of reads of these unclassified OTUs is stated as category "Unclassified".
Depending on the type of analysis, some taxonomic units might be removed as they to not match the expected clade, e.g. eukaryotes in a bacterial microbiome analysis. The number of removed reads is stated as category "Filtered". If this category is not listed, no filtering was performed.
A copy-number correction was performed for bacterial species only, see Angly FE et al. (2014). If the listed normalized fraction and raw fraction are identical, either no copy-number correction factor was available in the database or the factor is exactly one.

Sample Name (copy-number corrected read counts)

Taxonomic Level	Taxonomic Unit	Normalized Fraction	Raw Fraction
**A1.ITS1** (77,432 reads)			
s	**Pichia manshurica** (241 OTUs with 97-100% identity in 148-149bp to: Pichia manshurica)	**78.8%**	78.8%
s	**Pichia [Candida] ethanolica** (55 OTUs with 98-100% identity in 143bp to: [Candida] ethanolica)	**20.7%**	20.7%
g	**Pichia** (1 OTU with 100% identity in 164bp to: Pichia kudriavzevii, Pichia sp.)	**0.2%**	0.2%
s	**Aspergillus fumigatus** (1 OTU with 99% identity in 246bp to: Aspergillus fumigatus)	**0.1%**	0.1%
s	**Coniochaeta Lecythophora sp. TMS-2011** (1 OTU with 93% identity in 236bp to: Lecythophora sp. TMS-2011)	**0.1%**	0.1%
s	**Aspergillus sp. BF8** (1 OTU with 100% identity in 245bp to: Aspergillus sp. BF8)	**0.1%**	0.1%
g	**Trichosporon** (1 OTU with 100% identity in 185bp to: Trichosporon asahii, Trichosporon faecale, Trichosporon insectorum)	**0.0%**	0.0%
	**Other**	**0.0%**	0.0%
	**Unclassified** (1,933 reads)		
	**Filtered** (0 reads)		
**A1.ITS2** (158,289 reads)			
g	**Pichia** (154 OTUs with 98-100% identity in 259-276bp to: 5 unclassified Pichia strains, Pichia deserticola, Pichia manshurica, [Candida] ethanolica)	**84.4%**	84.4%
s	**Pichia manshurica** (38 OTUs with 99-100% identity in 276bp to: Pichia manshurica)	**15.4%**	15.4%
o	**Saccharomycetales** (1 OTU with 100% identity in 307bp to: Candida albicans, Pichia cecembensis, Pichia kudriavzevii, Pichia sp.)	**0.1%**	0.1%
g	**Coniochaeta** (1 OTU with 99% identity in 300bp to: Coniochaeta aff. decumbens IMUFRJ 52014, Lecythophora sp. TMS-2011)	**0.1%**	0.1%
	**Other**	**0.0%**	0.0%
	**Unclassified** (0 reads)		
	**Filtered** (0 reads)		
**A2.V1V3** (24,114 reads)			
s	**Prevotella maculosa** (18 OTUs with 86-88% identity in 492bp to: Prevotella maculosa)	**26.3%**	24.8%
s	**Acetobacter peroxydans** (4 OTUs with 99-100% identity in 436bp to: Acetobacter peroxydans)	**11.6%**	12.1%
s	**Prevotella oulorum** (10 OTUs with 84-88% identity in 276-491bp to: Prevotella oulorum)	**9.5%**	8.9%
s	**Prevotella sp. oral taxon 289** (6 OTUs with 82% identity in 276bp to: Prevotella sp. oral taxon 289)	**6.6%**	6.2%
s	**Atopobium sp. F0209** (2 OTUs with 88-92% identity in 276-468bp to: Atopobium sp. F0209)	**6.0%**	2.6%
s	**Lyticum sinuosum** (1 OTU with 79% identity in 459bp to: Lyticum sinuosum)	**4.7%**	1.7%

s	**Ruminiclostridium [Clostridium] cellulosi** (4 OTUs with 84-91% identity in 466-468bp to: [Clostridium] cellulosi)	**4.6%**	3.1%
s	**Clostridium sp. MB2-A37** (6 OTUs with 86-94% identity in 476bp to: Clostridium sp. MB2-A37)	**2.9%**	7.2%
g	**Prevotella** (4 OTUs with 82-86% identity in 276bp to: 2 unclassified Prevotella strains, Prevotella baroniae, Prevotella bryantii)	**2.7%**	2.6%
s	**Clostridium sp. strain S6** (7 OTUs with 89-92% identity in 471bp to: Clostridium sp. strain S6)	**2.2%**	5.5%
s	**Magnetococcus marinus** (1 OTU with 80% identity in 452bp to: Magnetococcus marinus)	**2.1%**	1.6%
g	**Brevundimonas** (1 OTU with 80% identity in 459bp to: 3 unclassified Brevundimonas strains)	**1.8%**	0.6%
s	**Swingsia samuiensis** (2 OTUs with 97% identity in 436bp to: Swingsia samuiensis)	**1.8%**	1.9%
s	**Prevotella timonensis** (4 OTUs with 87% identity in 491bp to: Prevotella timonensis)	**1.8%**	1.7%
s	**Clostridium sp. strain Z6** (4 OTUs with 86-94% identity in 276-471bp to: Clostridium sp. strain Z6)	**1.7%**	4.4%
g	**Dialister** (4 OTUs with 89% identity in 276-509bp to: Dialister micraerophilus, Dialister propionicifaciens, Dialister sp. oral taxon 119)	**1.6%**	3.7%
s	**Pectinodesmus pectinatus** (1 OTU with 80% identity in 276bp to: Pectinodesmus pectinatus)	**1.5%**	0.5%
s	**Prevotella sp. RZ** (3 OTUs with 85-86% identity in 276bp to: Prevotella sp. RZ)	**1.1%**	1.0%
s	**Intestinimonas sp. Marseille-P3083** (1 OTU with 87% identity in 484bp to: Intestinimonas sp. Marseille-P3083)	**1.0%**	1.6%
s	**cf. Oscillospira sp. BA04013493** (1 OTU with 86% identity in 276bp to: cf. Oscillospira sp. BA04013493)	**1.0%**	1.1%
o	**Bacteroidales** (1 OTU with 85% identity in 491bp to: Bacteroides plebeius, Prevotella oryzae)	**0.9%**	0.9%
s	**Gordonibacter faecihominis** (1 OTU with 89% identity in 464bp to: Gordonibacter faecihominis)	**0.8%**	0.4%
g	**Olsenella** (1 OTU with 93% identity in 470bp to: 2 unclassified Olsenella strains, Olsenella profusa)	**0.7%**	0.3%
s	**Gordonibacter sp. Marseille-P2775** (1 OTU with 87% identity in 463bp to: Gordonibacter sp. Marseille-P2775)	**0.6%**	0.3%
g	**Bacteroides** (2 OTUs with 84-85% identity in 494bp to: 2 unclassified Bacteroides strains, Bacteroides ovatus, Bacteroides plebeius, Bacteroides thetaiotaomicron)	**0.6%**	0.6%
s	**Dialister micraerophilus** (2 OTUs with 90% identity in 509bp to: Dialister micraerophilus)	**0.6%**	1.3%
s	**Monomorphina inconspicuus** (1 OTU with 78% identity in 276bp to: Monomorphina inconspicuus)	**0.5%**	0.2%
s	**Bacteroides sp. Marseille-P2644** (1 OTU with 83% identity in 496bp to: Bacteroides sp. Marseille-P2644)	**0.5%**	0.5%
s	**Prevotella paludivivens** (1 OTU with 84% identity in 276bp to: Prevotella paludivivens)	**0.4%**	0.4%
s	**Macellibacteroides fermentans** (1 OTU with 84% identity in 493bp to: Macellibacteroides fermentans)	**0.3%**	0.3%
s	**Prevotella sp. Marseille-P3114** (1 OTU with 81% identity in 276bp to: Prevotella sp. Marseille-P3114)	**0.3%**	0.3%
s	**Caloramator boliviensis** (1 OTU with 89% identity in 466bp to: Caloramator boliviensis)	**0.3%**	0.5%
s	**Bacteroides uniformis** (1 OTU with 84% identity in 491bp to: Bacteroides uniformis)	**0.2%**	0.3%
s	**Bacteroides caecigallinarum** (1 OTU with 81% identity in 276bp to: Bacteroides caecigallinarum)	**0.2%**	0.2%
s	**Clostridium hveragerdense** (1 OTU with 90% identity in 463bp to: Clostridium hveragerdense)	**0.2%**	0.5%
o	**Clostridiales** (1 OTU with 87% identity in 484bp to: Intestinimonas sp. Marseille-P3083, Oscillibacter sp. G2)	**0.1%**	0.2%
s	**Bacteroides sp. Marseille-P3132** (1 OTU with 82% identity in 276bp to: Bacteroides sp. Marseille-P3132)	**0.1%**	0.1%
s	**Megasphaera sueciensis** (1 OTU with 100% identity in 276bp to: Megasphaera sueciensis)	**0.1%**	0.2%
	**Other**	**0.0%**	0.0%
	**Unclassified** (405 reads)		
	**Filtered** (0 reads)		
**A3.V3V4** (49,323 reads)			
s	**Prevotella sp. oral taxon 300** (43 OTUs with 88-90% identity in 422bp to: Prevotella sp. oral taxon 300)	**30.2%**	30.4%
s	**Caproiciproducens galactitolivorans** (6 OTUs with 94-95% identity in 402bp to: Caproiciproducens galactitolivorans)	**16.7%**	11.9%
g	**Acetobacter** (5 OTUs with 99-100% identity in 402bp to: 2 unclassified Acetobacter strains, Acetobacter papayae, Acetobacter pasteurianus, Acetobacter peroxydans)	**6.7%**	7.5%
g	**Prevotella** (6 OTUs with 89-90% identity in 422bp to: 6 unclassified Prevotella strains, Prevotella albensis, Prevotella oris)	**6.7%**	6.8%
s	**Sporobacter termitidis** (2 OTUs with 94-95% identity in 405bp to: Sporobacter termitidis)	**5.6%**	4.0%
g	**Olsenella** (2 OTUs with 94-96% identity in 408bp to: 6 unclassified Olsenella strains, Olsenella profusa)	**5.5%**	2.5%

s	**Eubacterium coprostanoligenes** (4 OTUs with 90-91% identity in 405bp to: Eubacterium coprostanoligenes)	**5.1%**	9.4%
s	**Prevotella amnii** (4 OTUs with 89-90% identity in 422bp to: Prevotella amnii)	**5.0%**	5.0%
g	**Gluconobacter** (1 OTU with 98% identity in 402bp to: 6 unclassified Gluconobacter strains)	**2.3%**	1.7%
s	**Ethanoligenens harbinense** (1 OTU with 94% identity in 405bp to: Ethanoligenens harbinense)	**2.2%**	2.4%
s	**Dialister sp. S4-23** (3 OTUs with 92% identity in 427bp to: Dialister sp. S4-23)	**2.0%**	4.9%
s	**Prevotella sp. S8 F8** (1 OTU with 89% identity in 276bp to: Prevotella sp. S8 F8)	**1.8%**	1.8%
s	**Prevotella sp. Marseille-P2931** (1 OTU with 91% identity in 422bp to: Prevotella sp. Marseille-P2931)	**1.7%**	1.7%
s	**Denitrobacterium detoxificans** (2 OTUs with 91-93% identity in 401-402bp to: Denitrobacterium detoxificans)	**1.5%**	0.7%
s	**Ruminiclostridium [Clostridium] cellulosi** (4 OTUs with 92-96% identity in 405bp to: [Clostridium] cellulosi)	**1.1%**	0.8%
s	**Barnesiella viscericola** (1 OTU with 82% identity in 424bp to: Barnesiella viscericola)	**1.0%**	0.9%
o	**Clostridiales** (4 OTUs with 94-95% identity in 403-405bp to: Ethanoligenens harbinense, Linmingia china)	**0.9%**	1.4%
s	**Prevotella oryzae** (1 OTU with 88% identity in 422bp to: Prevotella oryzae)	**0.8%**	0.8%
s	**Prevotella sp. canine oral taxon 372** (1 OTU with 89% identity in 422bp to: Prevotella sp. canine oral taxon 372)	**0.4%**	0.4%
s	**Prevotella oris** (1 OTU with 91% identity in 422bp to: Prevotella oris)	**0.4%**	0.4%
s	**Prevotella sp. oral taxon F68** (1 OTU with 84% identity in 423bp to: Prevotella sp. oral taxon F68)	**0.4%**	0.4%
s	**Clostridium sp. MB2-A37** (2 OTUs with 95% identity in 402bp to: Clostridium sp. MB2-A37)	**0.3%**	0.8%
s	**Clostridium sp. X9** (2 OTUs with 93% identity in 402bp to: Clostridium sp. X9)	**0.3%**	0.8%
s	**Aminicella lysinilytica** (1 OTU with 97% identity in 404bp to: Aminicella lysinilytica)	**0.3%**	0.3%
s	**Clostridium sp. SW002** (1 OTU with 94% identity in 403bp to: Clostridium sp. SW002)	**0.3%**	0.7%
s	**Candidatus Symbiothrix dinenymphae** (1 OTU with 82% identity in 423bp to: Candidatus Symbiothrix dinenymphae)	**0.3%**	0.3%
s	**Vampirovibrio chlorellavorus** (1 OTU with 84% identity in 403bp to: Vampirovibrio chlorellavorus)	**0.2%**	0.2%
s	**Ralstonia solanacearum** (1 OTU with 89% identity in 404bp to: Ralstonia solanacearum)	**0.1%**	0.3%
s	**Clostridium sp. strain Z6** (1 OTU with 95% identity in 402bp to: Clostridium sp. strain Z6)	**0.1%**	0.4%
s	**Clostridium sp. strain S6** (2 OTUs with 92-93% identity in 402-405bp to: Clostridium sp. strain S6)	**0.1%**	0.3%
s	**Oxobacter sp. PPf50E4** (1 OTU with 92% identity in 402bp to: Oxobacter sp. PPf50E4)	**0.1%**	0.2%
s	**Caloramator quimbayensis** (1 OTU with 94% identity in 402bp to: Caloramator quimbayensis)	**0.1%**	0.1%
	**Other**	**0.0%**	0.0%
	**Unclassified** (0 reads)		
	**Filtered** (0 reads)		
**B1.ITS1** (52,770 reads)			
s	**Saccharomyces cerevisiae** (63 OTUs with 99-100% identity in 430bp to: Saccharomyces cerevisiae)	**65.6%**	65.6%
s	**Candida parapsilosis** (64 OTUs with 99-100% identity in 211bp to: Candida parapsilosis)	**17.4%**	17.4%
g	**Saccharomyces** (11 OTUs with 99-100% identity in 423bp to: Saccharomyces cerevisiae, Saccharomyces uvarum)	**10.5%**	10.5%
s	**Candida orthopsilosis** (12 OTUs with 99-100% identity in 202bp to: Candida orthopsilosis)	**5.9%**	5.9%
s	**Malassezia restricta** (1 OTU with 99% identity in 270bp to: Malassezia restricta)	**0.4%**	0.4%
s	**Pichia [Candida] ethanolica** (1 OTU with 100% identity in 143bp to: [Candida] ethanolica)	**0.2%**	0.2%
s	**Lachancea fermentati** (1 OTU with 100% identity in 275bp to: Lachancea fermentati)	**0.0%**	0.0%
	**Other**	**0.0%**	0.0%
	**Unclassified** (0 reads)		
	**Filtered** (0 reads)		
**B2.V1V3** (128,547 reads)			
g	**Allium** (77 OTUs with 92-100% identity in 433-438bp to: Allium cepa, Allium fistulosum, Allium macleanii, Allium nutans, Allium obliquum, Allium prattii, Allium sativum, Allium schoenoprasum, Allium victorialis)	**87.5%**	87.5%

o	**Asparagales** (9 OTUs with 99-100% identity in 276bp to: Agave, Allium, Aloe, Anemarrhena asphodeloides, Anthericum ramosum, Behnia reticulata, Beschorneria septentrionalis, Camassia scilloides, Chlorogalum pomeridianum, Chlorophytum rhizopendulum, Cypripedium macranthos, Echeandia sp. Steele 1101, Hesperaloe, Hesperocallis undulata, Hesperoyucca whipplei, Hosta, Maianthemum bicolor, Manfreda virginica, Milla biflora, Nolina atopocarpa, Oziroe biflora, Phormium tenax, Polygonatum, Schoenolirion croceum, Xanthorrhoea preissii, Yucca)	**6.5%**	6.5%
s	**Allium cepa** (8 OTUs with 99-100% identity in 481bp to: Allium cepa)	**6.0%**	6.0%
	**Other**	**0.0%**	0.0%
	**Unclassified** (0 reads)		
	**Filtered** (0 reads)		

Table 6: Condensed overview of the taxonomic composition of samples.

This table can be found as a file in the results directory. Please see the according section for details about result files.

4.2 Methods

As a first step of the microbiome analysis, all reads with ambiguous bases ("N") were removed. Chimeric reads were identified and removed based on the de-novo algorithm of UCHIME (Edgar RC et al., 2011) as implemented in the VSEARCH package (Rognes T et al., 2016).
The remaining set of high-quality reads was processed using minimum entropy decomposition (Eren AM, 2013 and 2015). Minimum Entropy Decomposition (MED) provides a computationally efficient means to partition marker gene datasets into OTUs (Operational Taxonomic Units). Each OTU represents a distinct cluster with significant sequence divergence to any other cluster. By employing Shannon entropy, MED uses only the information-rich nucleotide positions across reads and iteratively partitions large datasets while omitting stochastic variation. The MED procedure outperformes classical, identity based clustering algorithms. Sequences can be partitioned based on relevant single nucleotide differences without being susceptible to random sequencing errors. **This allows a decomposition of sequence data sets with a single nucleotide resultion.** Furthermore, the MED procedure identifies and filters random "noise" in the dataset, i.e. sequences with a very low abundance (less than $\approx$ 0.02% of the average sample size).
To assign taxonomic information to each OTU, DC-MEGABLAST alignments of cluster representative sequences to the sequence database were performed. A most specific taxonomic assignment for each OTU was then transferred from the set of best-matching reference sequences (lowest common taxonomic unit of all best hits). Hereby, a sequence identity of 70% accross at least 80% of the representative sequence was a minimal requirement for considering reference sequences.
Further processing of OTUs and taxonomic assignments was performed using the QIIME software package (version 1.9.1, http://qiime.org/). Abundances of bacterial taxonomic units were normalized using lineage-specific copy numbers of the relevant marker genes to improve estimates (Angly FE, 2014).

OTU-picking strategy: MED
Reference database: NCBI_nt (Release 2018-07-07)

References:

- **OTU picking:** Eren AM et al. (2013). Oligotyping: differentiating between closely related microbial taxa using 16s rRNA gene data. Methods Ecol Evol (4), 1111-1119.
  Eren AM et al. (2015) Minimum entropy decomposition: Unsupervised oligotyping for sensitive partitioning of high-throughput marker gene sequences. ISME Journal advance online publication, doi: 10.1038/ismej.2014.195.

- **Taxonomic assignment:** Altschul SF et al. (1990) Basic local alignment search tool. J Mol Biol 215(3), 403-410.

- **QIIME:** Caporaso JG et al. (2010) QIIME allows analysis of high-throughput community sequencing data. Nature Methods 7(5), 335-336.

- **Chimera detection:**
  Rognes T et al. (2016) VSEARCH: a versatile open source tool for metagenomics. PeerJ 4:e2584 https://doi.org/10.7717/peerj.2584.
  Edgar RC et al. (2011) UCHIME improves sensitivity and speed of chimera detection. Bioinformatics 27(16), 2194-2200.

- **Copy number correction:** Angly FE et al. (2014) CopyRighter: a rapid tool for improving the accuracy of microbial community profiles through lineage-specific gene copy number correction. Microbiome 2:11.

4.3 Output Files and Descriptions

The *MicrobiomeProfiling* directory contains the result files. All relevant files are described below. Some of these descriptions were excerpted from the official QIIME tutorials (http://qiime.org/tutorials/index.html).

01_Taxonomy_shortlist.txt: One of the **main results** of the microbiome analysis. This file can be used to get a quick overview of the microbiome. It contains a summarized list of indentified taxonomic units for each sample. The first two columns are the sample name and the total number of reads that where assigned to OTUs in this sample. The following columns list all taxonomic units with at least 0.0% of reads assigned to them. The individual columns state:

- The number of reads assigned to the taxonomic unit.
- The number of different OTUs that where classified as this taxonomic unit.
- The taxonomic level of the taxonomic unit. One of k...kingdom, p...phylum, c...class, o...order, f...family, g...genus, s...species.
- The abundancy-corrected fraction of reads assigned to the taxonomic unit.
- The fraction of reads assigned to the taxonomic unit.
- The identity and length of the best BLAST hit(s) to the database and a list of species that match with these alignment scores (not for all analysis types).

All taxonomic units with less than 0.0% of reads are collapsed in the category "Other". If the representative sequence of an OTU had no significant database match, no taxonomic unit could be assigned. The total number of reads of these unclassified OTUs is stated as category "Unclassified".
Depending on the type of analysis, some taxonomic units might be removed as they to not match the expected clade, e.g. eukaryotes in a bacterial microbiome analysis. The number of removed reads is stated as category "Filtered". If this category is not listed, no filtering was performed.
Please consider the provided identity and length of the best BLAST hits. The stated taxonomic unit was derived as lowest common ancestor of the best hits, but in case of a low sequence identity, it might be more appropriate to assign a higher taxonomic level than that of the lowest common ancestor.

02_Taxonomy_table.txt: One of the **main results** of the microbiome analysis. There is one line for each taxonomic unit and one column for each sample. The entries of the matrix are the estimated abundances of the respective taxonomic unit/sample combination. The file can be imported into Excel for further processing (sorting, calculations, diagrams).

03_OTU_representative_sequences.fasta: One of the **main results** of the microbiome analysis. Contains all read sequences of OTU representatives in FASTA format. The FASTA header contains the OTU identifier, the read identifier of the representative, the number of reads in the corresponding OTU, and the taxonomic classification. Representatives without taxonomic assignment are marked as "Unassigned", "Unclassified" or as "NOHIT", depending on the OTU picking method. Please note that representative sequences are not sample specific, i.e. a representative read subsumes similar reads of all samples. Thus, the given number of reads is the total number of reads of all samples that were assigned to the corresponding OTU.
Please note that OTUs only subsume sequences with identical lengths. Thus, OTU representatives may be prefixes of other OTU representatives. This occurs if assembled read pairs and (unassembled) single reads are processed together.

04_OTU_table.biom: One of the **main results** of the microbiome analysis. A file in BIOM format (http://biom-format.org/). This file is used as input by many QIIME scripts and is useful for downstream processing. OTUs of all samples are contained in this file.

05_OTU_table.txt: There is one line for each OTU and one column for each sample. The entries of the matrix are the estimated abundances of the respective OTU/sample combinations. The last column contains the taxonomic assignment of the OTU. OTUs without taxonomic assignment are marked as "Unassigned", "Unclassified", or "NOHIT", depending on the OTU picking method. Please see file `02_Taxonomy_table.txt` for the abundances per taxonomic unit and sample. The file can be imported into Excel for further processing (sorting, calculations, diagrams).

06_OTU_table_summary.txt: Contains a summary describing `05_OTU_table.txt`.

07_OTU_table_per_sample_statistics.txt: Contains statistics for each sample in `05_OTU_table.txt`.

08_Processed_reads.fasta.gz: Contains all read sequences in FASTA format that went into the OTU-picking process. Reads that were identified as chimeric are not contained in this file. Processed-read identifiers consist of the sample name and a sequential number, followed by the raw-read identifier and the length of the read. Reads of all samples are contained in this file.

09_OTU_read_assignment.txt: A mapping of OTU identifier to read identifier, i.e. each line represents one OTU, the first column contains the OTU identifier, all other columns contain the identifier of reads that are part of the OTU. OTUs/Reads of all samples are contained in this file.

10_Taxonomy_plots: This directory contains files `area_charts.html` and `bar_charts.html`. These files can be opened with any web browser. The data of `02_Taxonomy_table.txt` (as relative abundances) will be displayed as either area or bar chart plots. There are several plots, each for a different level of taxonomy: from phylum to species. Hereby, higher level plots give a more coarse-grained view on the data than lower level plots. Mouseover the plots to see which taxa are contributing to the percentage shown, and a click on the hyperlinks in the legend starts a web-search using the most specific taxonomic unit. Charts, legends, and tables can be exported by clicking on the respective hyperlinks.

Eurofins Genomics' products, services and applications reach the best quality and safety levels. They are carried out under strict QM and QA systems and comply with the following standards:

ISO 9001	Globally recognised as the standard quality management certification	GLP	The gold standard to conduct non-clinical safety studies
ISO 17025	Accredited analytical excellence	GCP	Pharmacogenomic services for clinical studies
ISO 13485	Oligonucleotides according to medical devices standard	cGMP	Products and testing according to pharma and biotech requirements

Eurofins Genomics GmbH • Anzinger Str. 7a • 85560 Ebersberg • Germany
